# Supplementary material for: Understanding the charge transfer effects of single atoms for boosting the performance of Na-S batteries
Source: Nat Commun. 2024 Apr 18;15:3325. doi: 10.1038/s41467-024-47628-3 (PMC11026416; doi:10.1038/s41467-024-47628-3)
Supplement: Supplementary file 1 — Supplementary Information [file 41467_2024_47628_MOESM1_ESM.pdf]

## Supporting Information

# Understanding the charge transfer effects of single atoms for boosting the performance of Na-S batteries

Yao-Jie Lei[1,2,8], Xinxin Lu[1,8], Hirofumi Yoshikawa[3], Daiju Matsumura[3], Yameng Fan[1], Lingfei Zhao[1], Jiayang Li[1], Shijian Wang[2], Qinfen Gu[4], Hua-Kun Liu[5], Shi-Xue Dou[5], Shanmukaraj Devaraj[6], Teofilo Rojo[7], Wei-Hong Lai[1]*, Michel Armand[6]*, Yun-Xiao Wang[5]*, Guoxiu Wang[2]*

[1]Institute for Superconducting & Electronic Materials, Australian Institute of Innovative Materials, University of Wollongong, Innovation Campus, Squires Way, North Wollongong, NSW 2500, Australia

[2]Centre for Clean Energy Technology, School of Mathematical and Physical Sciences, Faculty of Science, University of Technology Sydney, Sydney, NSW 2007, Australia

[3]School of Science and Technology, Kwansei Gakuin University, 2-1 Gakuen, Sanda, Hyogo, 669-1337 Japan

[4]Australian Synchrotron, 800 Blackburn Road, Clayton, Victoria 3168, Australia

[5]Institute of Energy Materials Science, University of Shanghai for Science and Technology, Shanghai, 200093 China

[6]Centre for Cooperative Research on Alternative Energies (CIC energiGUNE), Basque Research and Technology Alliance (BRTA). Alava Technology Park, Albert Einstein 48, 01510 Vitoria-Gasteiz, Spain

[7]Inorganic Chemistry Department, University of the Basque Country UPV/EHU, P.O. Box. 644, 48080 Bilbao, Spain

[8]These authors contributed equally: Yao-Jie Lei, Xinxin Lu

*E-mails: weihongl@uow.edu.au

marmand@cicenergigune.com

yunxiaowang@usst.edu.cn

Guoxiu.Wang@uts.edu.au

## Figures and Tables

**Table S1.** List of DFT-calculated and elemental features used as descriptors.

| Features | |
|---|---|
| **Period** | Period number of metal element |
| **RWIGS** | Bulk wigner-seitz radius of metal element |
| **Rm** | Atomic radius |
| **Nve** | Valence electron number of metal element |
| **Am** | Electron affinity |
| **Mm** | Atomic mass of metal element |
| **Xm** | Electronegativity of metal element |
| **Dm** | Density of metal element |
| **Ms** | Atomic mass of adsorption species |
| **Lms** | Length of metal-sufur bond |
| **D_Lmn** | Average change of metal-nitrogen bond length |

**Table S2**. Values of important 11 features through the RandomForest method over the 10 randomized data, and Pearson and Spearman methods.

| | Pearson | Spearman | RandomForest |
|---|---|---|---|
| Period | 0.077 | 0.15 | 0.005 |
| Group | 0.369 | 0.394 | 0.142 |
| RWIGS | 0.074 | 0.111 | 0.024 |
| Nve | 0.389 | 0.405 | 0.089 |
| Am | 0.22 | 0.293 | 0.019 |
| Mm | 0.118 | 0.278 | 0.016 |
| Xm | 0.052 | 0.137 | 0.015 |
| Dm | 0.093 | 0.163 | 0.014 |
| Ms | 0.577 | 0.58 | 0.203 |
| Lms | 0.502 | 0.639 | 0.412 |
| D_Lmn | 0.429 | 0.6 | 0.061 |

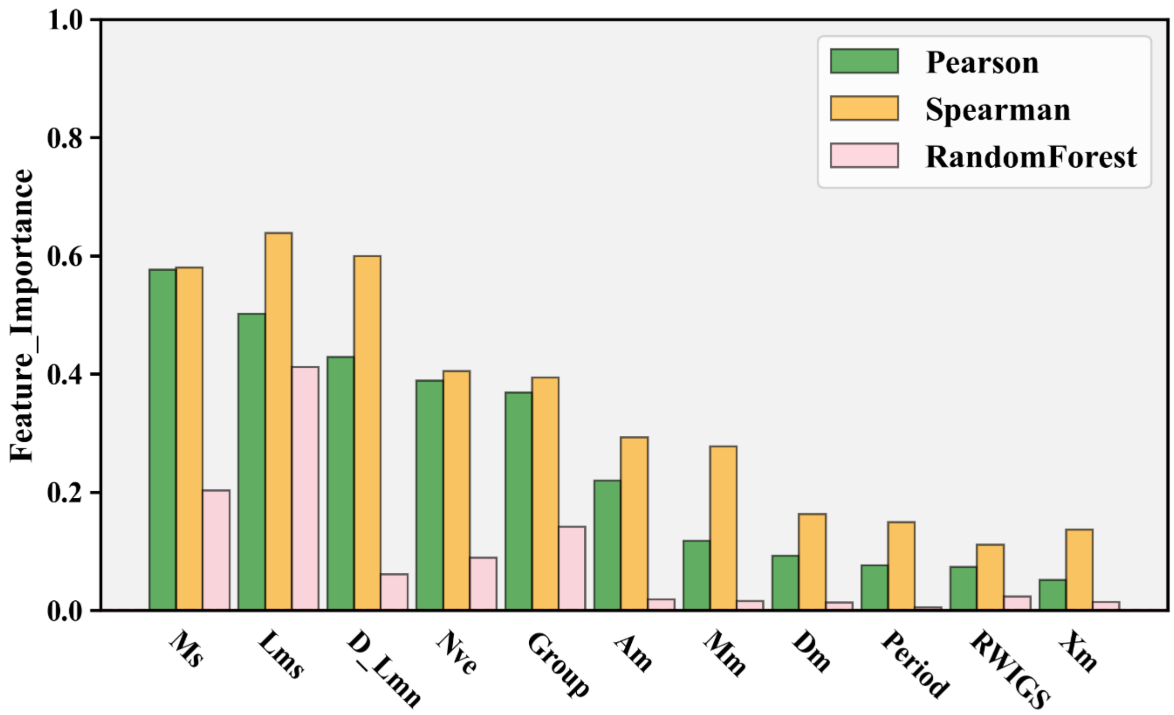


**Figure S1**. Importance of used 11 features through the RandomForest method over the 10 randomized data, and Pearson and Spearman methods.

The relationships between the used descriptors and adsorption activity were firstly analyzed through the Pearson correlation coefficient (Pearson), the Spearman correlation coefficient (Spearman) and the RandomForest feature importance (RandomForest) methods, as shown in Figure S1 and Table S2. The rankings of these descriptors by Pearson are basically consistent with that by Spearman, except for the metal-sulfur bond (Lms), whereas RandomForest method displays obviously distinction although the top two rankings are same with Spearman method. The rankings indicate that adsorption energies to polysulfides and sodium sulfide exhibits low correlations with most descriptors except the change of metal-nitrogen bond length (D_Lmn), Lms and valence electron number of metal element (Nve) and adsorbed species (Ms). After a rough screening of features according to the degree of feature importance, 11 main features (Figure S1) were chosen as descriptors. The *sklearn* package was employed to implement the data processing and import the ML algorithms.

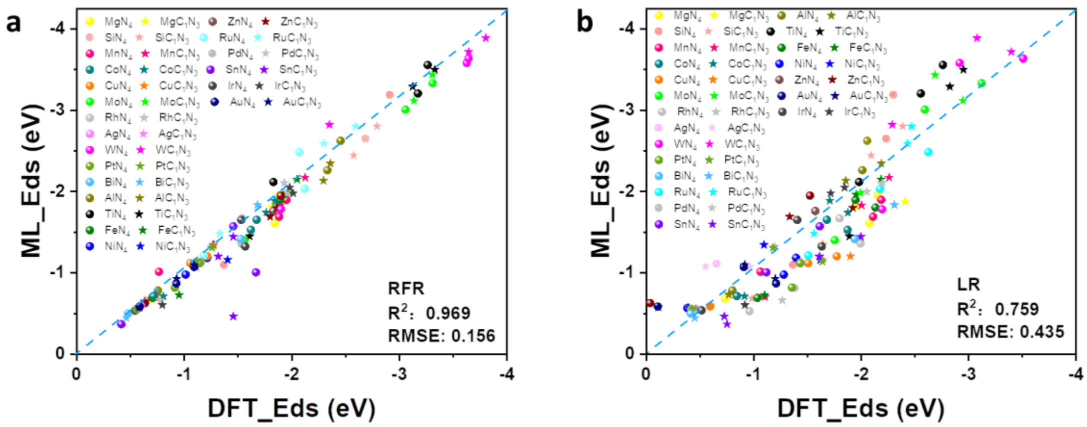


**Figure S2**. Comparison of the adsorption energy ($E_{ds}$) from the DFT calculations and the full-fit results using the various ML algorithms: (a) RFR and (b) LR, respectively. $R^2$: coefficient of determination; RMSE: root mean square error.

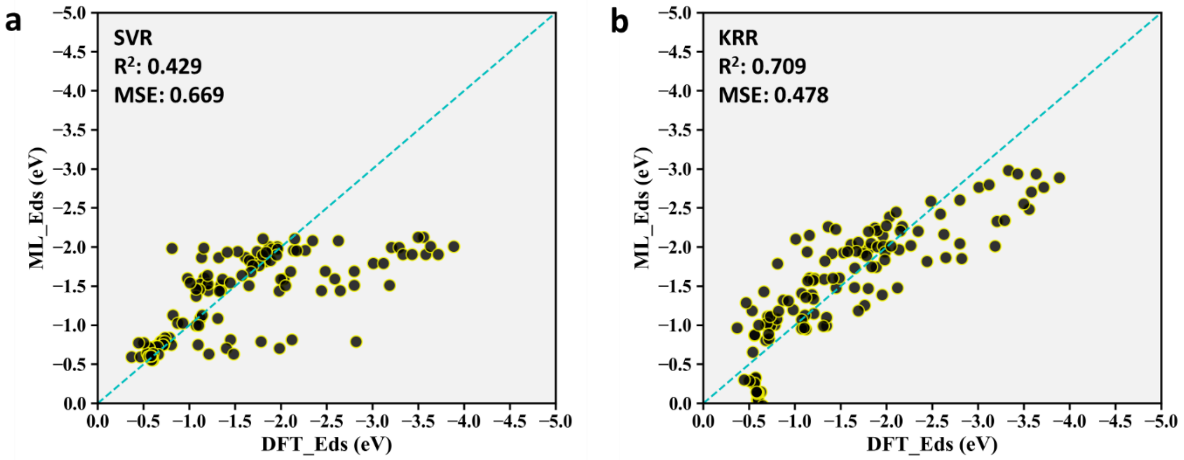


**Figure S3**. Predicted vs. DFT-calculated $E_{ds}$ of single-atom catalyst (SAC) materials by using SVR (a), and KRR (b) algorithms, with 80% training (blue dot) and 20% testing (red dot) data.

**Table S3**. Feature values for metal elements in ML modelling, including atomic number of metal element, period number of metal element, the group of metal element, bulk wigner-seitz

radius of metal element, valence electron number of metal element, atomic mass of metal element, electronegativity of metal element, density of metal element.

| Metal | Period | Group | RWIGS | Nve | Am | Mm | Xm | Dm | Ms | Lms | D_Lmn |
|---|---|---|---|---|---|---|---|---|---|---|---|
| $MgN_4$ | 3 | 2 | 2.88 | 2 | -40 | 24.03 | 1.31 | 1.74 | 256.48 | 2.773 | -0.03 |
| $MgN_4$ | 3 | 2 | 2.88 | 2 | -40 | 24.03 | 1.31 | 1.74 | 110.12 | 2.402 | -0.126 |
| $MgN_4$ | 3 | 2 | 2.88 | 2 | -40 | 24.03 | 1.31 | 1.74 | 78.06 | 2.62 | -0.112 |
| $MgCN_3$ | 3 | 2 | 2.88 | 2 | -40 | 24.03 | 1.31 | 1.74 | 256.48 | 2.705 | -0.046 |
| $MgCN_3$ | 3 | 2 | 2.88 | 2 | -40 | 24.03 | 1.31 | 1.74 | 110.12 | 2.511 | -0.105 |
| $MgCN_3$ | 3 | 2 | 2.88 | 2 | -40 | 24.03 | 1.31 | 1.74 | 78.06 | 2.414 | -0.159 |
| $AlN_4$ | 3 | 13 | 2.65 | 3 | 41.76 | 26.98 | 1.61 | 2.7 | 256.48 | 2.621 | -0.017 |
| $AlN_4$ | 3 | 13 | 2.65 | 3 | 41.76 | 26.98 | 1.61 | 2.7 | 110.12 | 2.24 | -0.084 |
| $AlN_4$ | 3 | 13 | 2.65 | 3 | 41.76 | 26.98 | 1.61 | 2.7 | 78.06 | 2.322 | -0.058 |
| $AlCN_3$ | 3 | 13 | 2.65 | 3 | 41.76 | 26.98 | 1.61 | 2.7 | 256.48 | 2.653 | -0.011 |
| $AlCN_3$ | 3 | 13 | 2.65 | 3 | 41.76 | 26.98 | 1.61 | 2.7 | 110.12 | 2.343 | -0.055 |
| $AlCN_3$ | 3 | 13 | 2.65 | 3 | 41.76 | 26.98 | 1.61 | 2.7 | 78.06 | 2.261 | -0.084 |
| $SiN_4$ | 3 | 14 | 2.48 | 4 | 134.06 | 28.08 | 1.9 | 2.33 | 256.48 | 2.184 | -0.071 |
| $SiN_4$ | 3 | 14 | 2.48 | 4 | 134.06 | 28.08 | 1.9 | 2.33 | 110.12 | 2.128 | -0.078 |
| $SiN_4$ | 3 | 14 | 2.48 | 4 | 134.06 | 28.08 | 1.9 | 2.33 | 78.06 | 2.213 | -0.057 |
| $SiCN_3$ | 3 | 14 | 2.48 | 4 | 134.06 | 28.08 | 1.9 | 2.33 | 256.48 | 2.657 | -0.008 |
| $SiCN_3$ | 3 | 14 | 2.48 | 4 | 134.06 | 28.08 | 1.9 | 2.33 | 110.12 | 2.228 | -0.052 |
| $SiCN_3$ | 3 | 14 | 2.48 | 4 | 134.06 | 28.08 | 1.9 | 2.33 | 78.06 | 2.148 | -0.073 |
| $TiN_4$ | 4 | 4 | 2.5 | 4 | 7.28 | 47.87 | 1.54 | 4.51 | 256.48 | 2.43 | -0.032 |

| | | | | | | | | | | | |
|---|---|---|---|---|---|---|---|---|---|---|---|
| $TiN_4$ | 4 | 4 | 2.5 | 4 | 7.28 | 47.87 | 1.54 | 4.51 | 110.12 | 2.261 | 0.028 |
| $TiN_4$ | 4 | 4 | 2.5 | 4 | 7.28 | 47.87 | 1.54 | 4.51 | 78.06 | 2.414 | 0.017 |
| $TiCN_3$ | 4 | 4 | 2.5 | 4 | 7.28 | 47.87 | 1.54 | 4.51 | 256.48 | 2.355 | -0.01 |
| $TiCN_3$ | 4 | 4 | 2.5 | 4 | 7.28 | 47.87 | 1.54 | 4.51 | 110.12 | 2.412 | -0.035 |
| $TiCN_3$ | 4 | 4 | 2.5 | 4 | 7.28 | 47.87 | 1.54 | 4.51 | 78.06 | 2.27 | -0.018 |
| $MnN_4$ | 4 | 7 | 2.5 | 7 | -50 | 54.94 | 1.55 | 7.21 | 256.48 | 2.656 | -0.008 |
| $MnN_4$ | 4 | 7 | 2.5 | 7 | -50 | 54.94 | 1.55 | 7.21 | 110.12 | 2.3 | -0.042 |
| $MnN_4$ | 4 | 7 | 2.5 | 7 | -50 | 54.94 | 1.55 | 7.21 | 78.06 | 2.388 | -0.023 |
| $MnCN_3$ | 4 | 7 | 2.5 | 7 | -50 | 54.94 | 1.55 | 7.21 | 256.48 | 2.556 | -0.013 |
| $MnCN_3$ | 4 | 7 | 2.5 | 7 | -50 | 54.94 | 1.55 | 7.21 | 110.12 | 2.28 | -0.018 |
| $MnCN_3$ | 4 | 7 | 2.5 | 7 | -50 | 54.94 | 1.55 | 7.21 | 78.06 | 2.218 | -0.032 |
| $FeN_4$ | 4 | 8 | 2.46 | 8 | 14.78 | 55.85 | 1.83 | 7.87 | 256.48 | 2.641 | -0.011 |
| $FeN_4$ | 4 | 8 | 2.46 | 8 | 14.78 | 55.85 | 1.83 | 7.87 | 110.12 | 2.213 | -0.013 |
| $FeN_4$ | 4 | 8 | 2.46 | 8 | 14.78 | 55.85 | 1.83 | 7.87 | 78.06 | 2.182 | -0.013 |
| $FeCN_3$ | 4 | 8 | 2.46 | 8 | 14.78 | 55.85 | 1.83 | 7.87 | 256.48 | 2.338 | -0.014 |
| $FeCN_3$ | 4 | 8 | 2.46 | 8 | 14.78 | 55.85 | 1.83 | 7.87 | 110.12 | 2.181 | -0.013 |
| $FeCN_3$ | 4 | 8 | 2.46 | 8 | 14.78 | 55.85 | 1.83 | 7.87 | 78.06 | 2.179 | -0.024 |
| $CoN_4$ | 4 | 9 | 2.46 | 9 | 63.89 | 58.93 | 1.88 | 8.86 | 256.48 | 2.447 | -0.025 |
| $CoN_4$ | 4 | 9 | 2.46 | 9 | 63.89 | 58.93 | 1.88 | 8.86 | 110.12 | 2.258 | -0.02 |
| $CoN_4$ | 4 | 9 | 2.46 | 9 | 63.89 | 58.93 | 1.88 | 8.86 | 78.06 | 2.244 | -0.022 |
| $CoCN_3$ | 4 | 9 | 2.46 | 9 | 63.89 | 58.93 | 1.88 | 8.86 | 256.48 | 2.26 | -0.033 |
| $CoCN_3$ | 4 | 9 | 2.46 | 9 | 63.89 | 58.93 | 1.88 | 8.86 | 110.12 | 2.235 | -0.025 |

| | | | | | | | | | | | |
|---|---|---|---|---|---|---|---|---|---|---|---|
| $CoCN_3$ | 4 | 9 | 2.46 | 9 | 63.89 | 58.93 | 1.88 | 8.86 | 78.06 | 2.259 | -0.023 |
| $NiN_4$ | 4 | 10 | 2.43 | 10 | 111.65 | 58.69 | 1.91 | 8.9 | 256.48 | 3.288 | -0.002 |
| $NiN_4$ | 4 | 10 | 2.43 | 10 | 111.65 | 58.69 | 1.91 | 8.9 | 110.12 | 2.502 | -0.012 |
| $NiN_4$ | 4 | 10 | 2.43 | 10 | 111.65 | 58.69 | 1.91 | 8.9 | 78.06 | 3.86 | 0 |
| $NiCN_3$ | 4 | 10 | 2.43 | 10 | 111.65 | 58.69 | 1.91 | 8.9 | 256.48 | 3.22 | -0.002 |
| $NiCN_3$ | 4 | 10 | 2.43 | 10 | 111.65 | 58.69 | 1.91 | 8.9 | 110.12 | 3.935 | -0.002 |
| $NiCN_3$ | 4 | 10 | 2.43 | 10 | 111.65 | 58.69 | 1.91 | 8.9 | 78.06 | 2.439 | -0.019 |
| $CuN_4$ | 4 | 11 | 2.2 | 11 | 119.23 | 63.55 | 1.9 | 8.96 | 256.48 | 3.276 | -0.001 |
| $CuN_4$ | 4 | 11 | 2.2 | 11 | 119.23 | 63.55 | 1.9 | 8.96 | 110.12 | 2.502 | -0.047 |
| $CuN_4$ | 4 | 11 | 2.2 | 11 | 119.23 | 63.55 | 1.9 | 8.96 | 78.06 | 3.77 | 0.001 |
| $CuCN_3$ | 4 | 11 | 2.2 | 11 | 119.23 | 63.55 | 1.9 | 8.96 | 256.48 | 3.281 | -0.001 |
| $CuCN_3$ | 4 | 11 | 2.2 | 11 | 119.23 | 63.55 | 1.9 | 8.96 | 110.12 | 2.615 | -0.023 |
| $CuCN_3$ | 4 | 11 | 2.2 | 11 | 119.23 | 63.55 | 1.9 | 8.96 | 78.06 | 2.471 | -0.034 |
| $ZnN_4$ | 4 | 12 | 2.4 | 12 | -58 | 65.38 | 1.65 | 7.14 | 256.48 | 2.79 | -0.019 |
| $ZnN_4$ | 4 | 12 | 2.4 | 12 | -58 | 65.38 | 1.65 | 7.14 | 110.12 | 2.266 | -0.151 |
| $ZnN_4$ | 4 | 12 | 2.4 | 12 | -58 | 65.38 | 1.65 | 7.14 | 78.06 | 2.327 | -0.104 |
| $ZnCN_3$ | 4 | 12 | 2.4 | 12 | -58 | 65.38 | 1.65 | 7.14 | 256.48 | 3.417 | -0.015 |
| $ZnCN_3$ | 4 | 12 | 2.4 | 12 | -58 | 65.38 | 1.65 | 7.14 | 110.12 | 2.376 | -0.104 |
| $ZnCN_3$ | 4 | 12 | 2.4 | 12 | -58 | 65.38 | 1.65 | 7.14 | 78.06 | 2.278 | -0.187 |
| $MoN_4$ | 5 | 6 | 2.75 | 6 | 72.1 | 95.96 | 2.16 | 10.28 | 256.48 | 2.2 | -0.023 |
| $MoN_4$ | 5 | 6 | 2.75 | 6 | 72.1 | 95.96 | 2.16 | 10.28 | 110.12 | 2.242 | -0.027 |
| $MoN_4$ | 5 | 6 | 2.75 | 6 | 72.1 | 95.96 | 2.16 | 10.28 | 78.06 | 2.375 | -0.106 |

| | | | | | | | | | | | |
|---|---|---|---|---|---|---|---|---|---|---|---|
| $MoCN_3$ | 5 | 6 | 2.75 | 6 | 72.1 | 95.96 | 2.16 | 10.28 | 256.48 | 2.284 | -0.077 |
| $MoCN_3$ | 5 | 6 | 2.75 | 6 | 72.1 | 95.96 | 2.16 | 10.28 | 110.12 | 2.359 | -0.107 |
| $MoCN_3$ | 5 | 6 | 2.75 | 6 | 72.1 | 95.96 | 2.16 | 10.28 | 78.06 | 2.245 | -0.009 |
| $RuN_4$ | 5 | 8 | 2.65 | 8 | 100.27 | 101.1 | 2.2 | 12.45 | 256.48 | 2.136 | -0.037 |
| $RuN_4$ | 5 | 8 | 2.65 | 8 | 100.27 | 101.1 | 2.2 | 12.45 | 110.12 | 2.294 | -0.009 |
| $RuN_4$ | 5 | 8 | 2.65 | 8 | 100.27 | 101.1 | 2.2 | 12.45 | 78.06 | 2.441 | -0.072 |
| $RuCN_3$ | 5 | 8 | 2.65 | 8 | 100.27 | 101.1 | 2.2 | 12.45 | 256.48 | 2.146 | -0.049 |
| $RuCN_3$ | 5 | 8 | 2.65 | 8 | 100.27 | 101.1 | 2.2 | 12.45 | 110.12 | 2.331 | -0.065 |
| $RuCN_3$ | 5 | 8 | 2.65 | 8 | 100.27 | 101.1 | 2.2 | 12.45 | 78.06 | 2.261 | -0.032 |
| $RhN_4$ | 5 | 9 | 2.65 | 9 | 100.27 | 102.9 | 2.28 | 12.41 | 256.48 | 3.078 | -0.002 |
| $RhN_4$ | 5 | 9 | 2.65 | 9 | 100.27 | 102.9 | 2.28 | 12.41 | 110.12 | 2.334 | -0.018 |
| $RhN_4$ | 5 | 9 | 2.65 | 9 | 100.27 | 102.9 | 2.28 | 12.41 | 78.06 | 4.118 | -0.005 |
| $RhCN_3$ | 5 | 9 | 2.65 | 9 | 100.27 | 102.9 | 2.28 | 12.41 | 256.48 | 2.304 | -0.038 |
| $RhCN_3$ | 5 | 9 | 2.65 | 9 | 100.27 | 102.9 | 2.28 | 12.41 | 110.12 | 2.319 | -0.03 |
| $RhCN_3$ | 5 | 9 | 2.65 | 9 | 100.27 | 102.9 | 2.28 | 12.41 | 78.06 | 2.341 | -0.023 |
| $PdN_4$ | 5 | 10 | 2.71 | 10 | 54.24 | 106.4 | 2.2 | 12.02 | 256.48 | 3.486 | -0.003 |
| $PdN_4$ | 5 | 10 | 2.71 | 10 | 54.24 | 106.4 | 2.2 | 12.02 | 110.12 | 2.728 | -0.006 |
| $PdN_4$ | 5 | 10 | 2.71 | 10 | 54.24 | 106.4 | 2.2 | 12.02 | 78.06 | 3.95 | -0.002 |
| $PdCN_3$ | 5 | 10 | 2.71 | 10 | 54.24 | 106.4 | 2.2 | 12.02 | 256.48 | 3.477 | -0.003 |
| $PdCN_3$ | 5 | 10 | 2.71 | 10 | 54.24 | 106.4 | 2.2 | 12.02 | 110.12 | 4.002 | -0.004 |
| $PdCN_3$ | 5 | 10 | 2.71 | 10 | 54.24 | 106.4 | 2.2 | 12.02 | 78.06 | 2.659 | -0.01 |
| $AgN_4$ | 5 | 11 | 2.84 | 11 | 125.86 | 107.9 | 1.93 | 10.49 | 256.48 | 3.443 | -0.005 |

| | | | | | | | | | | | |
|---|---|---|---|---|---|---|---|---|---|---|---|
| $AgN_4$ | 5 | 11 | 2.84 | 11 | 125.86 | 107.9 | 1.93 | 10.49 | 78.06 | 3.63 | -0.006 |
| $AgCN_3$ | 5 | 11 | 2.84 | 11 | 125.86 | 107.9 | 1.93 | 10.49 | 256.48 | 3.472 | -0.003 |
| $AgCN_3$ | 5 | 11 | 2.84 | 11 | 125.86 | 107.9 | 1.93 | 10.49 | 110.12 | 3.154 | -0.005 |
| $AgCN_3$ | 5 | 11 | 2.84 | 11 | 125.86 | 107.9 | 1.93 | 10.49 | 78.06 | 2.694 | -0.037 |
| $SnN_4$ | 5 | 14 | 2.96 | 4 | 107.29 | 118.7 | 1.96 | 7.26 | 256.48 | 3.866 | 0.002 |
| $SnN_4$ | 5 | 14 | 2.96 | 4 | 107.29 | 118.7 | 1.96 | 7.26 | 110.12 | 2.378 | 0.156 |
| $SnN_4$ | 5 | 14 | 2.96 | 4 | 107.29 | 118.7 | 1.96 | 7.26 | 78.06 | 4.024 | 0.027 |
| $SnCN_3$ | 5 | 14 | 2.96 | 4 | 107.29 | 118.7 | 1.96 | 7.26 | 256.48 | 2.516 | 0.058 |
| $SnCN_3$ | 5 | 14 | 2.96 | 4 | 107.29 | 118.7 | 1.96 | 7.26 | 110.12 | 2.481 | -0.035 |
| $SnCN_3$ | 5 | 14 | 2.96 | 4 | 107.29 | 118.7 | 1.96 | 7.26 | 78.06 | 5.535 | -0.029 |
| $WN_4$ | 6 | 6 | 2.75 | 6 | 78.76 | 183.8 | 2.36 | 19.3 | 256.48 | 2.139 | -0.055 |
| $WN_4$ | 6 | 6 | 2.75 | 6 | 78.76 | 183.8 | 2.36 | 19.3 | 110.12 | 2.236 | -0.036 |
| $WN_4$ | 6 | 6 | 2.75 | 6 | 78.76 | 183.8 | 2.36 | 19.3 | 78.06 | 2.355 | -0.128 |
| $WCN_3$ | 6 | 6 | 2.75 | 6 | 78.76 | 183.8 | 2.36 | 19.3 | 256.48 | 2.353 | -0.083 |
| $WCN_3$ | 6 | 6 | 2.75 | 6 | 78.76 | 183.8 | 2.36 | 19.3 | 110.12 | 2.328 | -0.142 |
| $WCN_3$ | 6 | 6 | 2.75 | 6 | 78.76 | 183.8 | 2.36 | 19.3 | 78.06 | 2.239 | -0.032 |
| $IrN_4$ | 6 | 9 | 2.84 | 9 | 150.94 | 192.2 | 2.2 | 22.56 | 256.48 | 3.392 | -0.002 |
| $IrN_4$ | 6 | 9 | 2.84 | 9 | 150.94 | 192.2 | 2.2 | 22.56 | 110.12 | 2.331 | -0.024 |
| $IrN_4$ | 6 | 9 | 2.84 | 9 | 150.94 | 192.2 | 2.2 | 22.56 | 78.06 | 4.2 | -0.007 |
| $IrCN_3$ | 6 | 9 | 2.84 | 9 | 150.94 | 192.2 | 2.2 | 22.56 | 256.48 | 2.259 | -0.045 |
| $IrCN_3$ | 6 | 9 | 2.84 | 9 | 150.94 | 192.2 | 2.2 | 22.56 | 110.12 | 2.316 | -0.041 |
| $IrCN_3$ | 6 | 9 | 2.84 | 9 | 150.94 | 192.2 | 2.2 | 22.56 | 78.06 | 2.337 | -0.031 |

| | | | | | | | | | | | |
|---|---|---|---|---|---|---|---|---|---|---|---|
| $PtN_4$ | 6 | 10 | 2.75 | 10 | 205.04 | 195.1 | 2.28 | 21.45 | 256.48 | 3.584 | -0.002 |
| $PtN_4$ | 6 | 10 | 2.75 | 10 | 205.04 | 195.1 | 2.28 | 21.45 | 110.12 | 2.69 | -0.009 |
| $PtN_4$ | 6 | 10 | 2.75 | 10 | 205.04 | 195.1 | 2.28 | 21.45 | 78.06 | 3.999 | -0.003 |
| $PtCN_3$ | 6 | 10 | 2.75 | 10 | 205.04 | 195.1 | 2.28 | 21.45 | 256.48 | 3.552 | -0.003 |
| $PtCN_3$ | 6 | 10 | 2.75 | 10 | 205.04 | 195.1 | 2.28 | 21.45 | 110.12 | 4.02 | -0.005 |
| $PtCN_3$ | 6 | 10 | 2.75 | 10 | 205.04 | 195.1 | 2.28 | 21.45 | 78.06 | 2.621 | -0.012 |
| $AuN_4$ | 6 | 11 | 2.84 | 11 | 222.75 | 197 | 2.54 | 19.32 | 256.48 | 3.542 | -0.002 |
| $AuN_4$ | 6 | 11 | 2.84 | 11 | 222.75 | 197 | 2.54 | 19.32 | 110.12 | 3.69 | -0.002 |
| $AuN_4$ | 6 | 11 | 2.84 | 11 | 222.75 | 197 | 2.54 | 19.32 | 78.06 | 3.063 | -0.004 |
| $AuCN_3$ | 6 | 11 | 2.84 | 11 | 222.75 | 197 | 2.54 | 19.32 | 256.48 | 3.53 | -0.003 |
| $AuCN_3$ | 6 | 11 | 2.84 | 11 | 222.75 | 197 | 2.54 | 19.32 | 110.12 | 3.664 | -0.003 |
| $AuCN_3$ | 6 | 11 | 2.84 | 11 | 222.75 | 197 | 2.54 | 19.32 | 78.06 | 3.084 | -0.003 |
| $BiN_4$ | 6 | 15 | 3.09 | 5 | 90.92 | 209 | 2.02 | 9.78 | 256.48 | 3.746 | 0.003 |
| $BiN_4$ | 6 | 15 | 3.09 | 5 | 90.92 | 209 | 2.02 | 9.78 | 78.06 | 3.015 | -0.08 |
| $BiCN_3$ | 6 | 15 | 3.09 | 5 | 90.92 | 209 | 2.02 | 9.78 | 256.48 | 3.721 | -0.004 |
| $BiCN_3$ | 6 | 15 | 3.09 | 5 | 90.92 | 209 | 2.02 | 9.78 | 78.06 | 2.679 | -0.146 |

**Table S4**. Eds from the DFT calculations and the full-fit results using the three ML algorithms, LR, RFR and GBR, respectively.

| Metal | Eds | LR-pred | RFR-pred | GBR-pred | SVR-pred | KRR-pred |
|---|---|---|---|---|---|---|
| $MgN_4$ | -0.684 | -0.732 | -0.702 | -0.714 | -0.793 | -0.997 |

| | | | | | | |
|---|---|---|---|---|---|---|
| $MgN_4$ | -1.6104 | -2.075 | -1.841 | -1.689 | -1.869 | -2.033 |
| $MgN_4$ | -1.9576 | -2.153 | -1.888 | -1.894 | -2.004 | -2.149 |
| $MgCN_3$ | -0.72 | -0.820 | -0.736 | -0.713 | -0.793 | -1.028 |
| $MgCN_3$ | -1.8096 | -1.958 | -1.811 | -1.793 | -1.869 | -1.986 |
| $MgCN_3$ | -1.8728 | -2.409 | -1.939 | -1.899 | -2.004 | -2.244 |
| $AlN_4$ | -0.7832 | -0.801 | -0.755 | -0.713 | -0.841 | -1.006 |
| $AlN_4$ | -2.2632 | -2.010 | -2.330 | -2.289 | -1.962 | -2.021 |
| $AlN_4$ | -2.624 | -2.055 | -2.453 | -2.554 | -2.080 | -2.165 |
| $AlCN_3$ | -0.7328 | -0.768 | -0.735 | -0.707 | -0.841 | -0.993 |
| $AlCN_3$ | -2.1344 | -1.856 | -2.294 | -2.236 | -1.962 | -1.970 |
| $AlCN_3$ | -2.344 | -2.188 | -2.358 | -2.443 | -2.080 | -2.203 |
| $SiN_4$ | -1.0936 | -1.366 | -1.370 | -1.134 | -0.749 | -0.990 |
| $SiN_4$ | -2.6448 | -2.236 | -2.689 | -2.626 | -1.445 | -1.867 |
| $SiN_4$ | -3.1856 | -2.303 | -2.915 | -3.062 | -1.513 | -2.015 |
| $SiCN_3$ | -0.68 | -0.987 | -0.675 | -0.699 | -0.749 | -0.809 |
| $SiCN_3$ | -2.44 | -2.096 | -2.579 | -2.487 | -1.444 | -1.819 |
| $SiCN_3$ | -2.7984 | -2.390 | -2.797 | -2.809 | -1.513 | -2.045 |
| $TiN_4$ | -2.1176 | -1.980 | -1.828 | -2.084 | -0.817 | -1.477 |
| $TiN_4$ | -3.2048 | -2.555 | -3.172 | -3.238 | -1.997 | -2.331 |
| $TiN_4$ | -3.556 | -2.761 | -3.263 | -3.456 | -2.128 | -2.486 |
| $TiCN_3$ | -1.4496 | -1.890 | -1.608 | -1.535 | -0.817 | -1.480 |
| $TiCN_3$ | -3.2888 | -2.824 | -3.124 | -3.196 | -1.997 | -2.341 |

| | | | | | | |
|---|---|---|---|---|---|---|
| $TiCN_3$ | -3.4976 | -2.951 | -3.329 | -3.395 | -2.128 | -2.554 |
| $MnN_4$ | -1.014 | -1.061 | -0.764 | -1.104 | -0.751 | -1.082 |
| $MnN_4$ | -1.6904 | -2.111 | -1.882 | -1.827 | -1.818 | -2.063 |
| $MnN_4$ | -1.8976 | -2.187 | -1.949 | -1.937 | -1.955 | -2.211 |
| $MnCN_3$ | -0.7032 | -1.103 | -0.721 | -0.730 | -0.751 | -1.114 |
| $MnCN_3$ | -1.8312 | -2.002 | -1.890 | -1.780 | -1.818 | -2.049 |
| $MnCN_3$ | -2.172 | -2.260 | -2.122 | -2.169 | -1.955 | -2.265 |
| $FeN_4$ | -0.6936 | -1.035 | -0.705 | -0.730 | -0.794 | -1.028 |
| $FeN_4$ | -1.8984 | -1.947 | -1.892 | -1.906 | -1.975 | -2.003 |
| $FeN_4$ | -1.8032 | -2.133 | -1.852 | -1.893 | -2.106 | -2.200 |
| $FeCN_3$ | -0.728 | -1.102 | -0.950 | -0.778 | -0.794 | -1.114 |
| $FeCN_3$ | -1.9608 | -1.952 | -1.921 | -1.904 | -1.975 | -2.012 |
| $FeCN_3$ | -2.1496 | -2.185 | -2.048 | -2.120 | -2.106 | -2.209 |
| $CoN_4$ | -0.7152 | -0.843 | -0.713 | -0.707 | -0.750 | -0.830 |
| $CoN_4$ | -1.6528 | -1.680 | -1.673 | -1.582 | -1.830 | -1.733 |
| $CoN_4$ | -1.5288 | -1.873 | -1.620 | -1.650 | -1.944 | -1.926 |
| $CoCN_3$ | -0.7128 | -0.914 | -0.805 | -0.725 | -0.751 | -0.887 |
| $CoCN_3$ | -1.8872 | -1.708 | -1.842 | -1.778 | -1.830 | -1.744 |
| $CoCN_3$ | -1.7408 | -1.875 | -1.762 | -1.799 | -1.944 | -1.923 |
| $NiN_4$ | -0.568 | -0.381 | -0.572 | -0.568 | -0.671 | -0.314 |
| $NiN_4$ | -1.1848 | -1.394 | -1.213 | -1.085 | -1.516 | -1.394 |
| $NiN_4$ | -0.9792 | -1.279 | -1.013 | -1.065 | -1.603 | -1.199 |

| | | | | | | |
|---|---|---|---|---|---|---|
| $NiCN_3$ | -0.5712 | -0.393 | -0.573 | -0.568 | -0.671 | -0.333 |
| $NiCN_3$ | -1.3448 | -1.094 | -1.268 | -1.253 | -1.515 | -0.993 |
| $NiCN_3$ | -1.1608 | -1.619 | -1.402 | -1.424 | -1.604 | -1.604 |
| $CuN_4$ | -0.588 | -0.594 | -0.592 | -0.594 | -0.641 | -0.045 |
| $CuN_4$ | -1.204 | -1.773 | -1.212 | -1.204 | -1.445 | -1.151 |
| $CuN_4$ | -1.12 | -1.505 | -1.055 | -1.076 | -1.528 | -0.952 |
| $CuCN_3$ | -0.5976 | -0.593 | -0.596 | -0.594 | -0.641 | -0.044 |
| $CuCN_3$ | -1.3448 | -1.641 | -1.277 | -1.244 | -1.445 | -1.101 |
| $CuCN_3$ | -1.2032 | -1.899 | -1.199 | -1.231 | -1.529 | -1.337 |
| $ZnN_4$ | -0.6288 | -0.034 | -0.635 | -0.618 | -0.729 | -0.149 |
| $ZnN_4$ | -1.7616 | -1.573 | -1.826 | -1.761 | -1.763 | -1.256 |
| $ZnN_4$ | -1.9504 | -1.523 | -1.896 | -1.910 | -1.899 | -1.389 |
| $ZnCN_3$ | -0.6488 | 0.095 | -0.630 | -0.663 | -0.729 | 0.026 |
| $ZnCN_3$ | -1.6904 | -1.333 | -1.797 | -1.700 | -1.763 | -1.188 |
| $ZnCN_3$ | -1.7992 | -1.921 | -1.863 | -1.834 | -1.899 | -1.470 |
| $MoN_4$ | -1.4016 | -1.753 | -1.556 | -1.433 | -0.706 | -1.919 |
| $MoN_4$ | -3.0064 | -2.592 | -3.057 | -3.130 | -1.793 | -2.767 |
| $MoN_4$ | -3.3312 | -3.120 | -3.309 | -3.306 | -1.905 | -2.982 |
| $MoCN_3$ | -1.9824 | -1.992 | -1.979 | -1.930 | -0.706 | -1.940 |
| $MoCN_3$ | -3.1168 | -2.947 | -3.135 | -3.163 | -1.793 | -2.800 |
| $MoCN_3$ | -3.432 | -2.688 | -3.306 | -3.311 | -1.905 | -2.939 |
| $RuN_4$ | -1.2136 | -1.506 | -1.181 | -1.227 | -0.634 | -1.598 |

| | | | | | | |
|---|---|---|---|---|---|---|
| $RuN_4$ | -2.032 | -2.174 | -2.119 | -2.069 | -1.593 | -2.388 |
| $RuN_4$ | -2.484 | -2.625 | -2.070 | -2.537 | -1.692 | -2.587 |
| $RuCN_3$ | -1.4832 | -1.561 | -1.328 | -1.397 | -0.634 | -1.605 |
| $RuCN_3$ | -2.5888 | -2.430 | -2.298 | -2.497 | -1.593 | -2.424 |
| $RuCN_3$ | -2.8 | -2.469 | -2.593 | -2.626 | -1.692 | -2.604 |
| $RhN_4$ | -0.5304 | -0.960 | -0.539 | -0.570 | -0.627 | -1.187 |
| $RhN_4$ | -1.3616 | -1.993 | -1.531 | -1.576 | -1.587 | -2.260 |
| $RhN_4$ | -1.6704 | -1.799 | -1.537 | -1.645 | -1.685 | -1.948 |
| $RhCN_3$ | -0.6616 | -1.265 | -0.777 | -0.716 | -0.627 | -1.429 |
| $RhCN_3$ | -1.9968 | -2.052 | -1.958 | -1.960 | -1.587 | -2.274 |
| $RhCN_3$ | -2.1048 | -2.197 | -1.929 | -1.873 | -1.686 | -2.450 |
| $PdN_4$ | -0.5568 | -0.462 | -0.561 | -0.573 | -0.720 | -0.874 |
| $PdN_4$ | -1.132 | -1.436 | -1.161 | -1.096 | -1.868 | -1.941 |
| $PdN_4$ | -0.8104 | -1.383 | -0.913 | -0.887 | -1.986 | -1.790 |
| $PdCN_3$ | -0.5608 | -0.463 | -0.563 | -0.573 | -0.720 | -0.877 |
| $PdCN_3$ | -1.3192 | -1.202 | -1.270 | -1.292 | -1.867 | -1.590 |
| $PdCN_3$ | -1.1568 | -1.649 | -1.128 | -1.091 | -1.988 | -2.150 |
| $AgN_4$ | -0.6 | 0.324 | -0.596 | -0.583 | -0.557 | -0.137 |
| $AgN_4$ | -1.1088 | -0.656 | -1.102 | -1.073 | -1.461 | -1.130 |
| $AgCN_3$ | -0.5856 | 0.338 | -0.590 | -0.564 | -0.557 | -0.127 |
| $AgCN_3$ | -1.0744 | -0.555 | -1.113 | -1.075 | -1.375 | -1.072 |
| $AgCN_3$ | -1.0728 | -0.967 | -1.112 | -1.125 | -1.461 | -1.412 |

| | | | | | | |
|---|---|---|---|---|---|---|
| $SnN_4$ | -0.3696 | -0.751 | -0.414 | -0.429 | -0.595 | -0.965 |
| $SnN_4$ | -1.0072 | -1.118 | -1.666 | -1.037 | -1.544 | -2.104 |
| $SnN_4$ | -1.5736 | -1.614 | -1.456 | -1.523 | -1.640 | -1.945 |
| $SnCN_3$ | -0.4656 | -0.726 | -1.456 | -0.484 | -0.596 | -1.289 |
| $SnCN_3$ | -1.4432 | -1.996 | -1.455 | -1.423 | -1.544 | -2.231 |
| $SnCN_3$ | -1.2 | -1.610 | -1.315 | -1.229 | -1.638 | -1.576 |
| $WN_4$ | -1.7816 | -2.195 | -1.898 | -1.819 | -0.792 | -1.892 |
| $WN_4$ | -3.58 | -2.916 | -3.627 | -3.616 | -1.906 | -2.706 |
| $WN_4$ | -3.6336 | -3.508 | -3.642 | -3.649 | -2.007 | -2.936 |
| $WCN_3$ | -2.8208 | -2.289 | -2.349 | -2.801 | -0.792 | -1.856 |
| $WCN_3$ | -3.7168 | -3.397 | -3.645 | -3.657 | -1.906 | -2.767 |
| $WCN_3$ | -3.8864 | -3.078 | -3.803 | -3.851 | -2.007 | -2.890 |
| $IrN_4$ | -0.54 | -0.515 | -0.543 | -0.552 | -0.640 | -0.655 |
| $IrN_4$ | -1.3256 | -1.633 | -1.567 | -1.416 | -1.439 | -1.820 |
| $IrN_4$ | -1.6512 | -1.405 | -1.530 | -1.620 | -1.507 | -1.481 |
| $IrCN_3$ | -0.6048 | -0.917 | -0.795 | -0.668 | -0.641 | -1.001 |
| $IrCN_3$ | -1.9784 | -1.715 | -2.013 | -1.874 | -1.439 | -1.838 |
| $IrCN_3$ | -2.0504 | -1.846 | -1.981 | -2.023 | -1.508 | -2.012 |
| $PtN_4$ | -0.5528 | -0.414 | -0.556 | -0.568 | -0.602 | -0.251 |
| $PtN_4$ | -1.12 | -1.432 | -1.144 | -1.101 | -1.090 | -1.358 |
| $PtN_4$ | -0.8208 | -1.354 | -0.912 | -0.893 | -1.128 | -1.182 |
| $PtCN_3$ | -0.5584 | -0.424 | -0.557 | -0.568 | -0.602 | -0.261 |

| | | | | | | |
|---|---|---|---|---|---|---|
| $PtCN_3$ | -1.3096 | -1.178 | -1.256 | -1.305 | -1.089 | -0.990 |
| $PtCN_3$ | -1.1392 | -1.639 | -1.113 | -1.096 | -1.128 | -1.567 |
| $AuN_4$ | -0.5872 | -0.106 | -0.584 | -0.553 | -0.610 | -0.143 |
| $AuN_4$ | -1.0728 | -0.907 | -1.091 | -1.067 | -1.000 | -0.959 |
| $AuN_4$ | -0.8712 | -1.208 | -0.925 | -0.984 | -1.029 | -1.320 |
| $AuCN_3$ | -0.58 | -0.112 | -0.581 | -0.553 | -0.610 | -0.148 |
| $AuCN_3$ | -1.1 | -0.916 | -1.090 | -1.067 | -1.000 | -0.967 |
| $AuCN_3$ | -0.9288 | -1.199 | -0.928 | -0.984 | -1.029 | -1.314 |
| $BiN_4$ | -0.5 | -0.413 | -0.475 | -0.473 | -0.774 | -0.292 |
| $BiN_4$ | -1.4152 | -1.940 | -1.526 | -1.481 | -1.936 | -1.603 |
| $BiCN_3$ | -0.4464 | -0.450 | -0.461 | -0.473 | -0.774 | -0.304 |
| $BiCN_3$ | -1.836 | -2.309 | -1.680 | -1.806 | -1.936 | -1.749 |

After analyzing the importance of features, the predicted performance of the five ML algorithms was evaluated, in which SVR and KRR algorithms show distinct overfitting with relatively large or zero RMSE (Figure S3a and S3b). Consequently, the other three algorithms were mainly used to observe the fitting results, and it was found that GBR algorithm (vs. RFR and LR algorithm, Figure 1e, Figure S2a, S2b and Table S3, Table S4) gives a much better fitting. Then, the dataset was split into training and learning sets over the same 20 repeated and randomized data for ML prediction. Similar with fitting results, LR algorithm shows a poor prediction, with the $R^2$ and RMSE values of the training/testing sets are 0.759 and 0.435 eV, respectively (Supplementary information, Figure S4a). The testing set with RFR algorithm exhibits a better prediction performance than the training set, with the $R^2$ of 0.969 and RMSE

of 0.156 eV (Supplementary information, Figure S4b). It may indicate that the predicted Eds values present a deviation from the actual Eds values and prediction of RFR overestimated Eds. Corresponding to a better fitting result, GBR algorithm presents a relatively accurate prediction with the $R^2$ and RMSE values of the training/testing sets are 0.97 and 0.153 eV, respectively (Supplementary information, Figure S4c). The average errors of RMSE and MSE for LR, RFR and GBR algorithms over 20 times of random training and learning are shown in Figure S5 and Table S5. Both RFR and GBR algorithms give a lower error bar of RMSE/MSE than LR algorithm, consistent with the model prediction results. According to the prediction in Figure 1e, a linear relationship between the adsorption energy Eads and diverse SACs is obtained, which demonstrates that the prediction results of ML are like those of DFT calculations. Meanwhile, compared to DFT calculations, machine learning can accelerate the collection of large numbers of computational results.

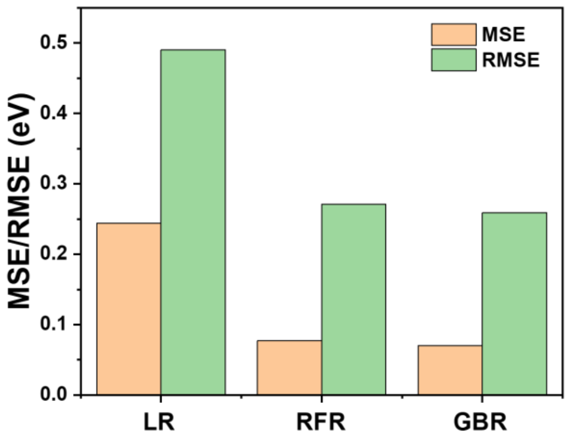


**Figure S4**. Average MSE/RMSE values of LR, RFR, and GBR over the same 20 repeated and randomized data, respectively.

**Table S5**. The average MSE/RMSE value of LR, RFR and GBR over the same 20 repeated and randomized data.

| | MSE | RMSE |
|---|---|---|
| LR | 0.244 | 0.490 |
| RFR | 0.077 | 0.271 |
| GBR | 0.070 | 0.259 |

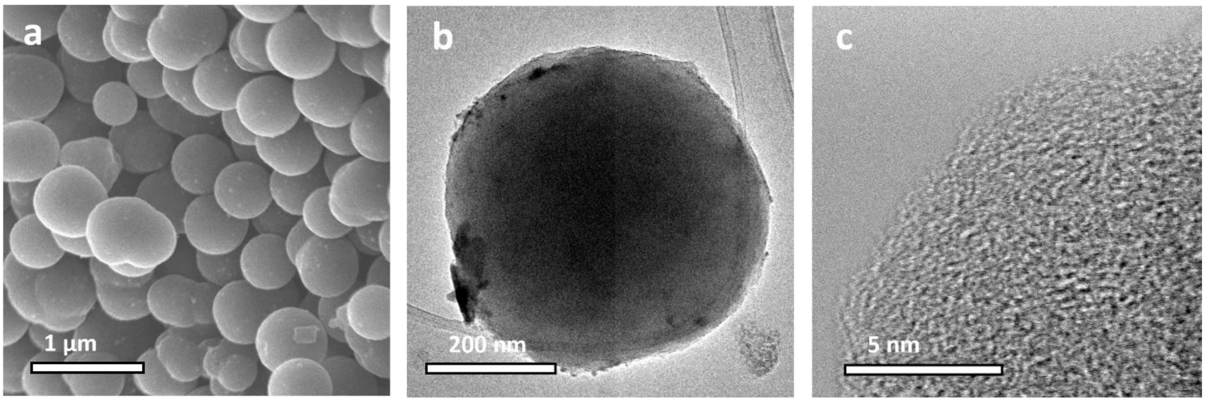


**Figure S5**. **The structural information of S@$Mn_1$-PNC**. (a) SEM image of S@$Mn_1$-PNC nanospheres. (b) TEM image of S@$Mn_1$-PNC. (c) High-resolution TEM (HRTEM) image of S@$Mn_1$-PNC nanospheres in different areas.

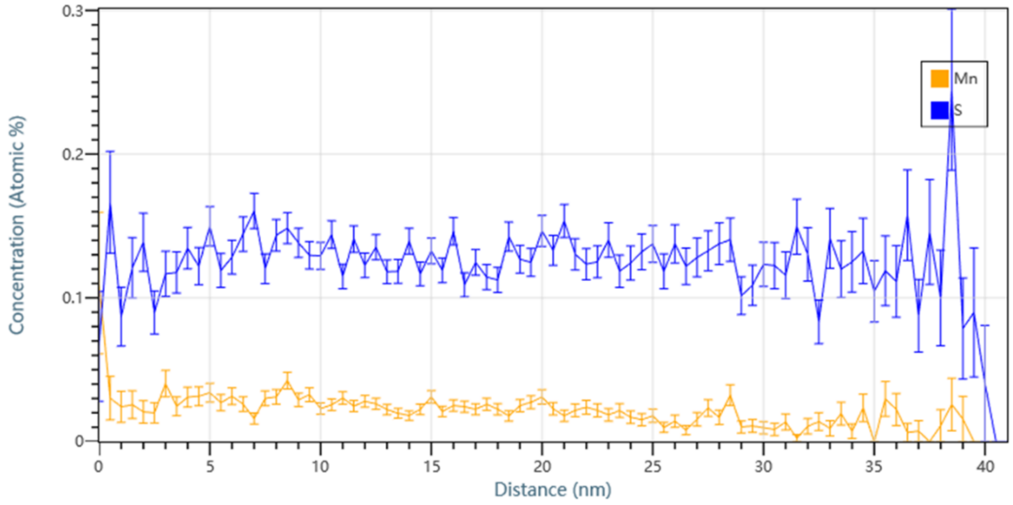


**Figure S6**. Proximity histogram across the Mn and S iso-concentration surface.

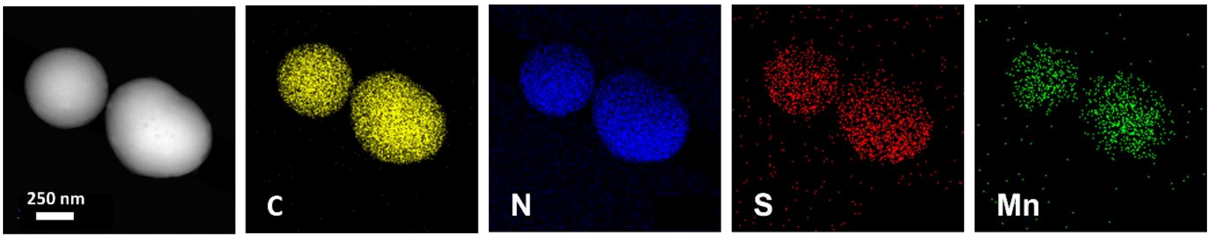


**Figure S7**. Element mappings of S@$Mn_1$-PNC nanospheres.

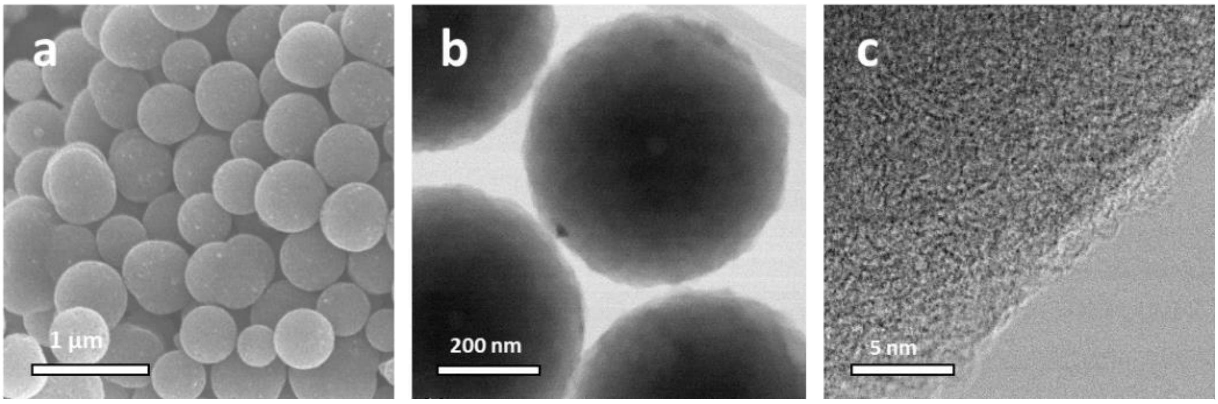

**Figure S8**. **The structural information of S@Ni$_1$-PNC**. (a) SEM image of S@Ni$_1$-PNC nanospheres. (b) The TEM image of S@Ni$_1$-PNC. (c) The high-resolution TEM (HRTEM) image of S@Ni$_1$-PNC nanospheres in different areas.

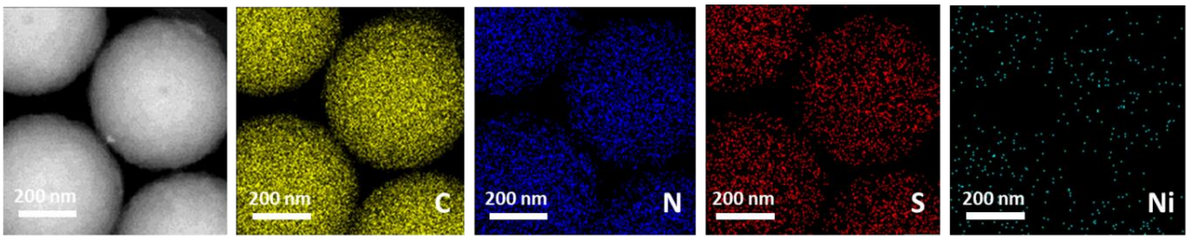


**Figure S9**. Element mappings of S@Ni$_1$-PNC nanospheres.

**Table S6.** EXAFS fitting parameters at the Mn K-edge for various samples（$S_0^2$=0.92）

| Samples | shell | CN | R(Å) | $\sigma^2$ | $\Delta E_0$ | R factor |
|---|---|---|---|---|---|---|
| MnO | Mn-O | 6 | 2.20 | 0.0097 | -1.7 | 0.0030 |
| | Mn-Mn | 12 | 3.14 | 0.0095 | | |
| S@Mn$_1$-PNC | Mn-N | 4.3 | 2.18 | 0.0081 | 2.8±1.9 | 0.0157 |

$S_0^2$ is the amplitude reduction factor; $^a N$: coordination numbers; $^b R$: bond distance; $^c\sigma^2$: Debye-Waller factors; $^d$ $\Delta E_0$: the inner potential correction. *R* factor: goodness of fit.

The obtained XAFS data was processed in Athena (version 0.9.26) for background, pre-edge line and post-edge line calibrations. Then Fourier transformed fitting was carried out in Artemis (version 0.9.26). The $k^3$ weighting, k-range of 3 - 12 Å$^{-1}$ and R range of 1 - ~3 Å were used for the fitting of MnO; k-range of 3 - 10 Å$^{-1}$ and R range of 1 - ~2 Å were used for the fitting of samples. The four parameters, coordination number, bond length, Debye-Waller factor and $E_0$ shift (CN, R, $\Delta E_0$) were fitted without anyone was fixed, the $\sigma^2$ was set.

For Wavelet Transform analysis, the χ(k) exported from Athena was imported into the Hama Fortran code. The parameters were listed as follow: R range, 1 - 4 Å, k range, 0 - 11 $Å^{-1}$ for samples; k weight, 3; and Morlet function with κ=10, σ=1 was used as the mother wavelet to provide the overall distribution.

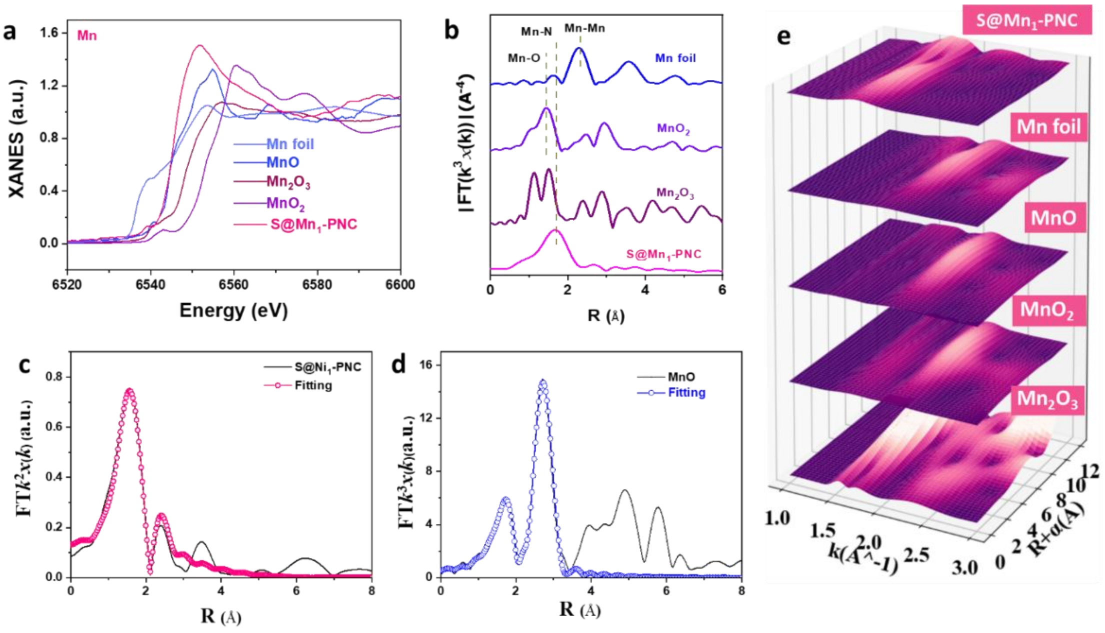


Figure S10. The electronic environment of S@Mn1-PNC. (a) X-ray absorption near-edge (XANES) spectra of the Mn K-edge of the S@Mn1-PNC nanospheres, MnO, $MnO_2$, $Mn_2O_3$, and Mn foil. (b) Analysis of the EXAFS spectra of the S@Mn1-PNC nanospheres. (c) The corresponding EXAFS fitting curves of the S@Mn1-PNC nanospheres in *R* space. (d) The corresponding EXAFS fitting curves of MnO in *R* space. (e) Wavelet transforms of EXAFS spectra for S@Mn1-PNC nanospheres and reference samples.

**Table S7**. EXAFS fitting parameters at the Fe K-edge for various samples（path amplitude, $S_0^2$ = 0.81）

| Sample | shell | CN | R(Å) | $\sigma^2$ | $\Delta E_0$ (eV) | R factor |
|---|---|---|---|---|---|---|
| Fe foil | Fe-Fe | 8 | 2.47 | 0.0051 | 6.8 | 0.0034 |
| | Fe-Fe | 6 | 2.85 | 0.0065 | | |
| S@Fe$_1$-PNC | Fe-N | 4.1 | 1.95 | 0.0075 | 10.5 | 0.0169 |

$S_0^2$ is the amplitude reduction factor; $^aN$: coordination number; $^bR$: bond length; $^c\sigma^2$: Debye-Waller factor; $^d$ $\Delta E_0$: the inner potential correction. *R* factor: goodness of fit.

The obtained XAFS data were processed in Athena (version 0.9.26) for background, pre-edge line, and post-edge line calibrations. Then, the Fourier transformed fitting was carried out in Artemis (version 0.9.26). $k^3$ weighting, the *k*-range of 3 – 14 Å$^{-1}$, and the *R* range of 1 - ~3 Å were used for the fitting of Fe foil; the *k*-range of 3 – 11 Å$^{-1}$ and *R* range of 1 - ~3 Å were used for the fitting of samples. The four parameters: coordination number, bond length, Debye-Waller factor, and $E_0$ shift (CN, *R*, $\Delta E_0$) were fitted without any one of them fixed, but the $\sigma^2$ was set.

For wavelet transform analysis, the $\chi(k)$ exported from Athena was imported into the Hama Fortran code. The parameters are listed as follows: *R* range, 1 – 4 Å, *k* range, 0 – 11 Å$^{-1}$ for samples; *k* weight, 3; and the Morlet function with $\kappa$ = 10, $\sigma$ = 1 was used as the mother wavelet to provide the overall distribution.

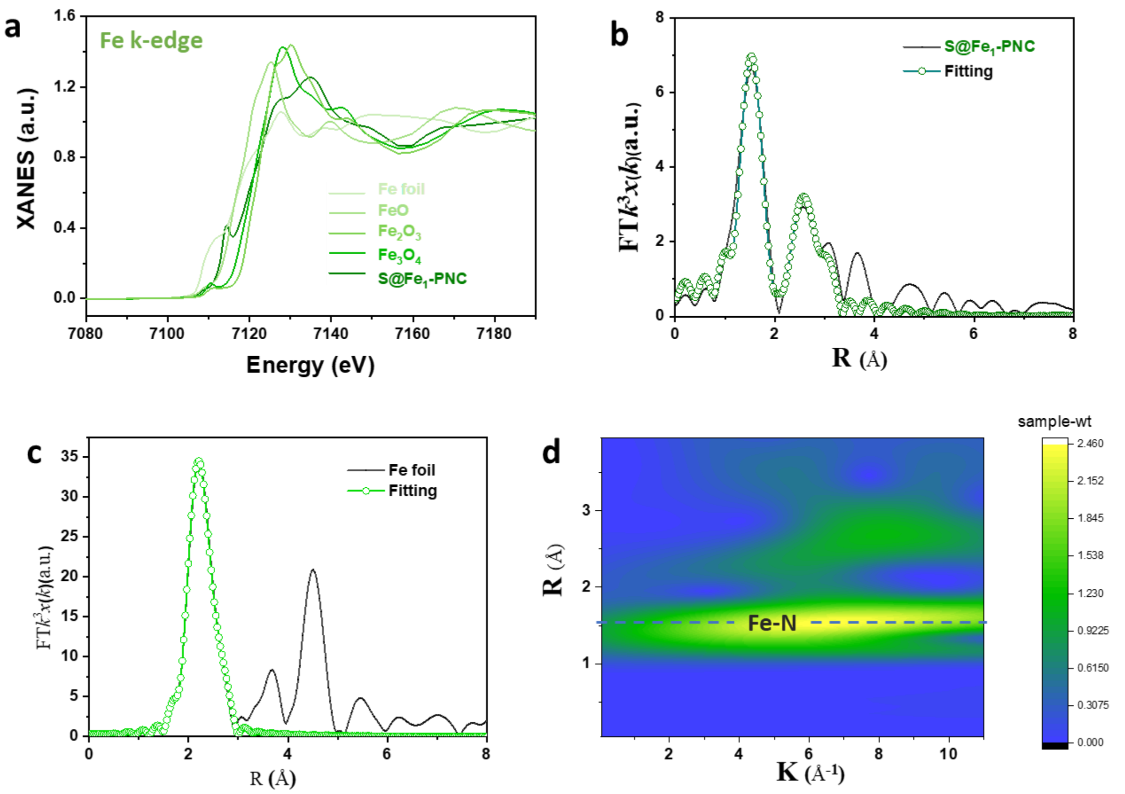


**Figure S11**. **The electronic environment of S@Fe₁-PNC**. (a) XANES spectra of the Fe K-edge of the S@Fe₁-PNC nanospheres, FeO sample, $Fe_2O_3$ sample, $Fe_3O_4$ sample, and Fe foil. (b) The fitting in *R* space of S@Fe₁-PNC nanospheres. (c) The corresponding EXAFS fitting curves of Fe foil in R space. (d) Wavelet transform of EXAFS spectrum for S@Fe₁-PNC nanospheres.

**Table S8.** EXAFS fitting parameters at the Ni K-edge for various samples（$S_0^2$=0.84）

| Samples | shell | CN | R(Å) | $\sigma^2$ | $\Delta E_0$ | R factor |
|---|---|---|---|---|---|---|
| Ni foil | Ni-Ni | 12 | 2.48 | 0.0061 | 7.0 | 0.0012 |
| S@$Ni_1$-PNC | Ni-N | 3.8 | 2.01 | 0.0099 | 6.8 | 0.0093 |

$S_0^2$ is the amplitude reduction factor; $^aN$: coordination numbers; $^bR$: bond distance; $^c\sigma^2$: Debye-Waller factors; $^d$ $\Delta E_0$: the inner potential correction. *R* factor: goodness of fit.

The obtained XAFS data was processed in Athena (version 0.9.26) for background, pre-edge line and post-edge line calibrations. Then Fourier transformed fitting was carried out in Artemis (version 0.9.26). The $k^3$ weighting, k-range of 3 - 12 $Å^{-1}$ and R range of 1 - ~3 Å were used for the fitting of Ni foil; k-range of 3 - 10 $Å^{-1}$ and R range of 1 - ~3 Å were used for the fitting of samples. The four parameters, coordination number, bond length, Debye-Waller factor and $E_0$ shift (CN, R, $\Delta E_0$) were fitted without anyone was fixed, the $\sigma^2$ was set.

For Wavelet Transform analysis, the $\chi(k)$ exported from Athena was imported into the Hama Fortran code. The parameters were listed as follow: R range, 1 - 4 Å, k range, 0 - 11 $Å^{-1}$ for samples; k weight, 3; and Morlet function with $\kappa$=10, $\sigma$=1 was used as the mother wavelet to provide the overall distribution.

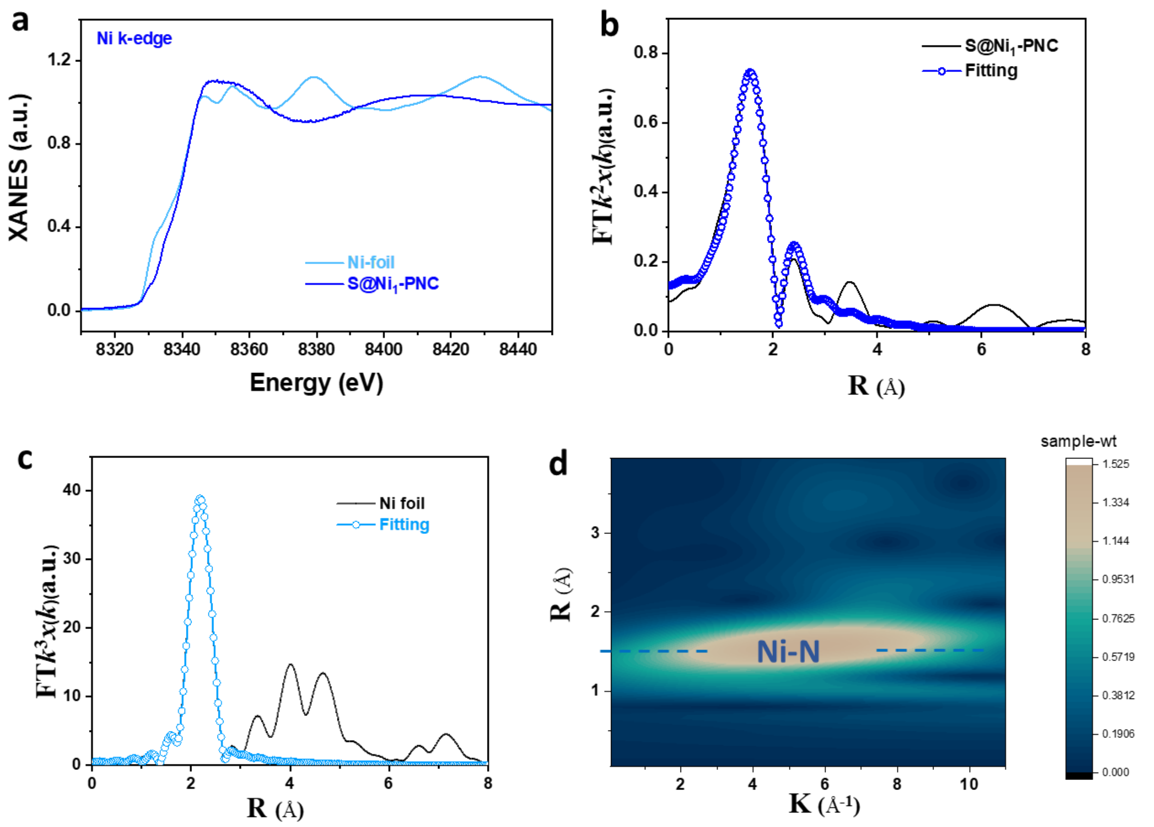


**Figure S12**. **The electronic environment of S@Ni₁-PNC**. (a) XANES spectra of the Ni K-edge of S@$Ni_1$-PNC nanospheres and Ni foil. (b) The EXAFS fitting curves of S@$Ni_1$-PNC nanospheres in R space. (c) The corresponding EXAFS fitting curves of Ni foil in R space. (d) Wavelet transform of EXAFS spectrum for S@$Ni_1$-PNC nanospheres.

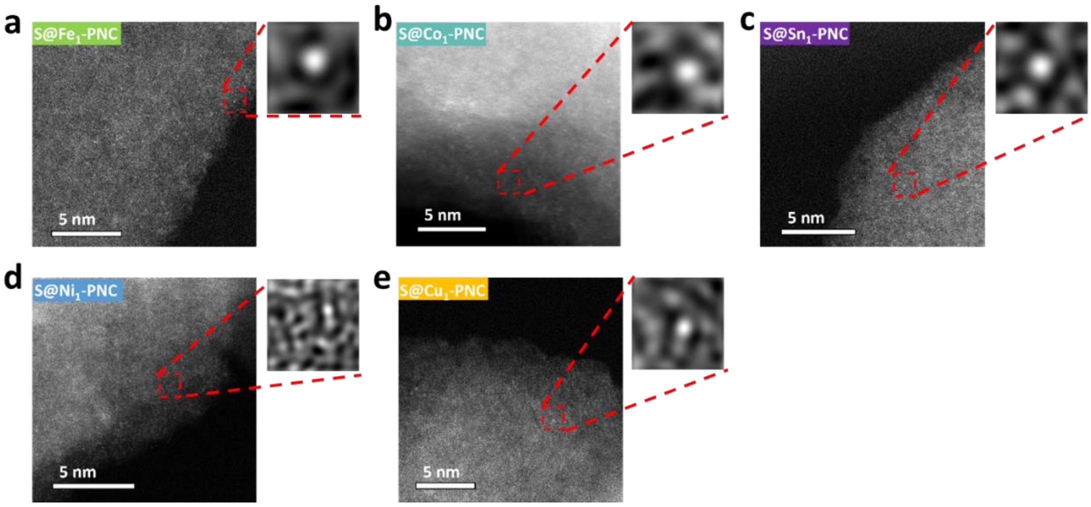


**Figure S13**. High-resolution HAADF images of (a) S@$Fe_1$-PNC, (b) S@$Co_1$-PNC, (c) S@$Sn_1$-PNC, (d) S@$Ni_1$-PNC, and (e) S@$Cu_1$-PNC with the corresponding fast Fourier transform (FFT) inversed HRTEM images of the selected red rectangle areas.

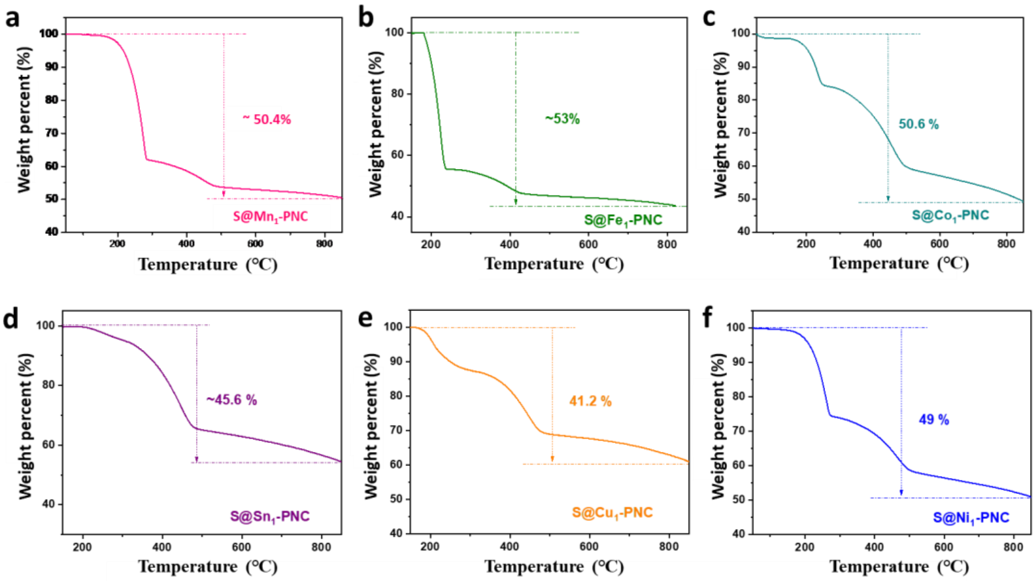


**Figure S14**. Thermogravimetric analysis (TGA) curves of (a) S@$Mn_1$-PNC, (b) S@$Fe_1$-PNC, (c) S@$Co_1$-PNC, (d) S@$Sn_1$-PNC, (e) S@$Cu_1$-PNC, and (f) S@$Ni_1$-PNC.

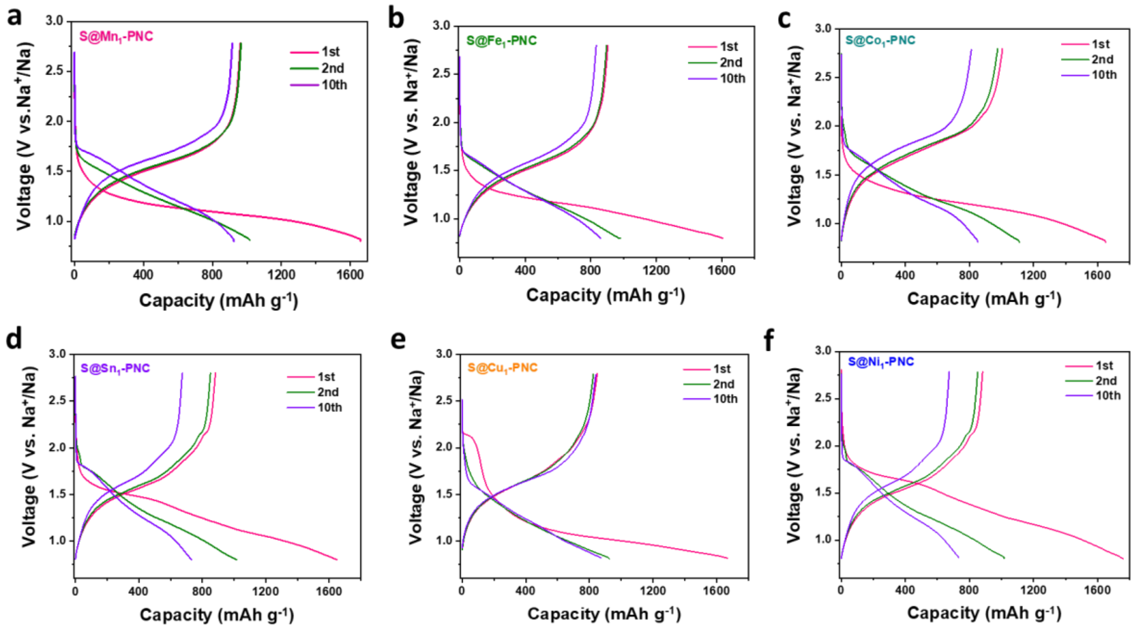


**Figure S15**. Charge-discharge curves of the selected cycles for (a) S@Mn1-PNC, (b) S@Fe1-PNC, (c) S@Co1-PNC, (d) S@Sn1-PNC, (e) S@Cu1-PNC, (f) S@Ni1-PNC.

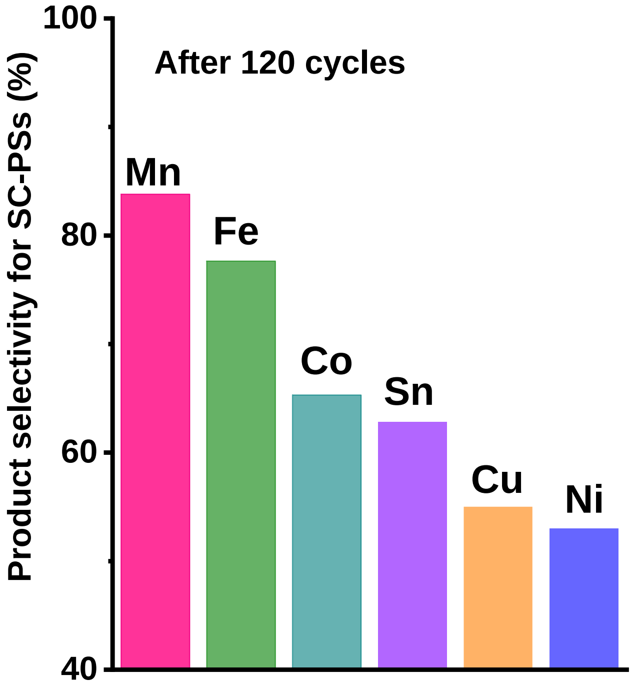


**Figure S16**. The percentages of short-chain polysulfides for S@Mn1-PNC, S@Fe1-PNC, S@Co1-PNC, S@Sn1-PNC, S@Cu1-PNC, and S@Ni1-PNC in the initial charges.

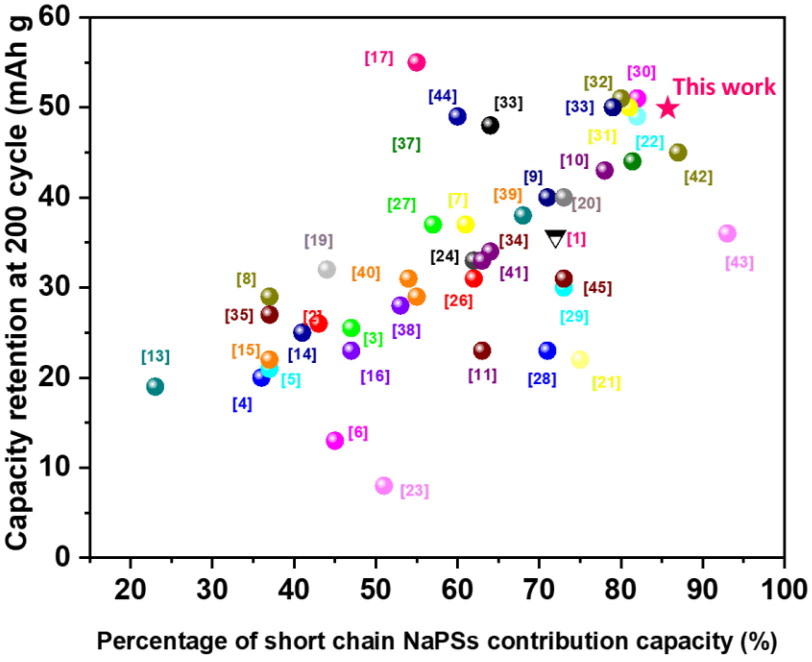


**Figure S17**. Short-chain product-selectivity in the first cycle vs. capacity retention after 200 cycles for S@Mn1-PNC compared with previous counterparts.

**Table S9.** Comparison of electrochemical performances of this work with previous studies.

| No. | Cathodic materials | Electrolytes | Short-chain contribution ratio in the first cycle (%) | Retention ratio after 200 cycles (%) | Rate performances | Cycling performances | Ref. |
|---|---|---|---|---|---|---|---|
| [1] | $FeS_2$@NCMS/S composites | 1M $NaClO_4$ in EC/PC+5%FEC | 72 | 35.7 | 139 mAh $g^{-1}$/ 5A | Retained 524 mAh $g^{-1}$ after 300 cycles at 0.1 A $g^{-1}$ | 7 |
| [2] | S@$Co_n$-HC | 1M $NaClO_4$ in EC/PC+5%FEC | 43 | 26 | 220 mA h $g^{-1}$/ 5A | Retained 508 mA h $g^{-1}$ after 600 cycles at 0.1 A $g^{-1}$ | 8 |
| [3] | S@HC | 1M $NaClO_4$ in EC/PC+5%FEC | 47 | 25.5 | 180 mA h $g^{-1}$/ 5A | Retained 367 mA h $g^{-1}$ after 600 cycles at 0.1 A $g^{-1}$ | |

| | | | | | | | |
|---|---|---|---|---|---|---|---|
| [4] | S@$Fe_n$-HC | 1M $NaClO_4$ in EC/PC+5%FEC | 36 | 20 | 220 mAh $g^{-1}$/ 5A | Retained 394 mAh $g^{-1}$ after 1000 cycles at 0.1 A $g^{-1}$ | 9 |
| [5] | S@$Ni_n$-HC | 1M $NaClO_4$ in EC/PC+5%FEC | 37 | 21 | 140 mAh $g^{-1}$/ 5A | Retained 263 mAh$g^{-1}$ after 1000 cycles at 0.1 A $g^{-1}$ | |
| [6] | S@$Cu_n$-HC | 1M $NaClO_4$ in EC/PC+5%FEC | 45 | 13 | 56 mAh $g^{-1}$/ 5A | Retained 263 mAh$g^{-1}$ after 1000 cycles at 0.1 A $g^{-1}$ | |
| [7] | ZCS | 1M NaClO4 in EC/PC+5%FEC | 61 | 37 | 188 mAh $g^{-1}$/5.0 A | 250 mAh $g^{-1}$ after 2000 cycles at 1 A $g^{-1}$ | 10 |
| [8] | ZnS | 1M NaClO4 in EC/PC+5%FEC | 37 | 29 | 116 mAh $g^{-1}$/5.0 A | 188 mAh $g^{-1}$ after 2000 cycles at 1 A $g^{-1}$ | |
| [9] | $CoS_2$ | 1M NaClO4 in EC/PC+5%FEC | 71 | 40 | 82 mAh $g^{-1}$/5.0 A | 66 mAh $g^{-1}$ after 2000 cycles at 1 A $g^{-1}$ | |
| [10] | S@$AgS_2$@HNCS | 1M $NaClO_4$ in EC/PC+3%FEC | 78 | 43 | 275 mAh $g^{-1}$/ 2.0A | Retained 391 mAh $g^{-1}$ after 1,600 cycles at 0.5 A g-1 | 11 |
| [11] | S@HNCS | 1M $NaClO_4$ in EC/PC+3%FEC | 63 | 23 | 62 mAh $g^{-1}$/ 2.0A | Retained 530 mAh g-1 after 200 cycles at 0.1 A g-1 | |
| [12] | $Fe_1$@NC@S | 1M $NaClO_4$ in EC/PC+5%FEC | 54 | 31 | 205 mAh$g^{-1}$/ 10.0 A | 455 mAh$g^{-1}$ after1320 cycles at 0.3 A$g^{-1}$ | 12 |
| [13] | $Ru_1$@NC@S | 1M $NaClO_4$ in EC/PC+5%FEC | 23 | 19 | 180 mAh$g^{-1}$/ 10.0 A | 699 mAh$g^{-1}$ after1320 cycles at 0.3 A$g^{-1}$ | |
| [14] | $Mn_1$@NC@S | 1M $NaClO_4$ in EC/PC+5%FEC | 41 | 25 | 112 mAh$g^{-1}$/ 10.0 A | 281 mAh$g^{-1}$ after1320 cycles at 0.3 A$g^{-1}$ | |
| [15] | $Ge_1$@NC@S | 1M $NaClO_4$ in EC/PC+5%FEC | 37 | 22 | 42 mAh$g^{-1}$/10.0 A | 281 mAh $g^{-1}$ after1320 cycles at 0.3 A $g^{-1}$ | |
| [16] | $Ni_1$@NC@S | 1M $NaClO_4$ in EC/PC+5%FEC | 47 | 23 | 117 mAh$g^{-1}$/10.0 A | 469 mAh $g^{-1}$ after1320 cycles at 0.3 A $g^{-1}$ | |

| | | | | | | | |
|---|---|---|---|---|---|---|---|
| [17] | MMPCS-800 | 1M $NaClO_4$ in EC/PC+5%FEC | 55 | 55 | 470 $mAhg^{-1}$/5.0 A | 420 mAh $g^{-1}$ after 2000 cycles at 2 A $g^{-1}$ | [12] |
| [18] | MMPCS-700 | 1M $NaClO_4$ in EC/PC+5%FEC | 69 | 43 | 410 $mAhg^{-1}$/5.0 A | 580 mAh $g^{-1}$ after 500 cycles at 0.5 A $g^{-1}$ | |
| [19] | MMPCS-900 | 1M $NaClO_4$ in EC/PC+5%FEC | 44 | 32 | 281 $mAhg^{-1}$/5.0 A | 573 mAh $g^{-1}$ after 500 cycles at 0.5 A $g^{-1}$ | |
| [20] | 300s | 1M $NaClO_4$ in EC/PC+5%FEC | 73 | 40 | 243 $mAhg^{-1}$/1.0 A | 535 mAh $g^{-1}$ after 500 cycles at 0.1 Ag-1 | [13] |
| [21] | 155s | 1M $NaClO_4$ in EC/PC+5%FEC | 75 | 22 | 142 $mAhg^{-1}$/1.0 A | 279 mAhg-1 after 500 cycles at 0.1 A $g^{-1}$ | |
| [22] | $Co_1$-ZnS/C | 1M $NaClO_4$ in EC/PC+5%FEC | 82 | 49 | 390 $mAhg^{-1}$/ 3.0 A | 640 mAh $g^{-1}$ after 500 cycles at 0.1 A $g^{-1}$ | [14] |
| [23] | SPAN cathode with FEC | 1 M $NaClO_4$ in TEGDME with 10 vol % FEC | 62 | 33 | 409 $mAhg^{-1}$/ 1.0 A | 587 mAh $g^{-1}$ after 200 cycles at 0.2 A $g^{-1}$ | [15] |
| [24] | SPAN cathode without FEC | 1 M $NaClO_4$ in TEGDME | 51 | 8 | 170 $mAhg^{-1}$/ 1.0 A | ~0 mAh $g^{-1}$ after 200 cycles at 0.2 A $g^{-1}$ | |
| [25] | S@NPC-700 | 1M $NaClO_4$ in EC/DEC | 64 | 48 | 280 $mAhg^{-1}$/ 3.3 A | 418.9 mAh $g^{-1}$ after 200 cycles at 0.8 A $g^{-1}$ | [16] |
| [26] | S@NPC-650 | 1M $NaClO_4$ in EC/DEC | 62 | 37 | 149 $mAhg^{-1}$/ 3.3 A | 209.7 mAh $g^{-1}$ after 200 cycles at 0.8 A $g^{-1}$ | |
| [27] | S@NPC-750 | 1M $NaClO_4$ in EC/DEC | 57 | 31 | 73 $mAhg^{-1}$/ 3.3 A | 101.3 mAh $g^{-1}$ after 200 cycles at 0.8 A $g^{-1}$ | |
| [28] | S@iMCHS | 1 M $NaClO_4$ in TEGDME with 10 vol % FEC | 71 | 23 | 127 $mAhg^{-1}$/ 5 A | 292 mAh $g^{-1}$ after 200 cycles at 0.1 A $g^{-1}$ | [17] |
| [29] | Sugar-derived | 1M $NaPF_6$ + 0.25M $NaNO_3$ in a TEGDME | 73 | 30 | 370 $mAhg^{-1}$/ 1.67 A | 370 mAh $g^{-1}$ after 1500 cycles at 1.67 A $g^{-1}$ | [18] |

| | | | | | | | |
|---|---|---|---|---|---|---|---|
| [30] | S@CNT-Co@NC-0.25 | 1M $NaClO_4$ in EC/PC+2%FEC | 82 | 51 | 474.2 $mAhg^{-1}$/ 5 A | 634.6 mAh $g^{-1}$ after 120 cycles at 0.2 A $g^{-1}$ | [19] |
| [31] | S@NC | 1M $NaClO_4$ in EC/PC+2%FEC | 81 | 50 | 263.8 $mAhg^{-1}$/ 5 A | 547.9 mAh $g^{-1}$ after 120 cycles at 0.2 A $g^{-1}$ | |
| [32] | S@CNT-Co@NC-0.5 | 1M $NaClO_4$ in EC/PC+2%FEC | 80 | 51 | 368 $mAhg^{-1}$/ 5 A | 568.4 mAh $g^{-1}$ after 120 cycles at 0.2 A $g^{-1}$ | |
| [33] | S@CNT-Co@NC-0.75 | 1M $NaClO_4$ in EC/PC+2%FEC | 79 | 50 | 287.2 $mAhg^{-1}$/ 5 A | 474.7 mAh $g^{-1}$ after 120 cycles at 0.2 A $g^{-1}$ | |
| [34] | S@Co@NC | 1M $NaClO_4$ in EC/PC+2%FEC | 64 | 34 | 62.8 $mAhg^{-1}$/ 5 A | 216 mAh $g^{-1}$ after 120 cycles at 0.2 A $g^{-1}$ | |
| [35] | PCMs | 1M $NaClO_4$ in EC/PC+5%FEC | 37 | 27 | 56 $mAhg^{-1}$/ 2 A | 290 mAh $g^{-1}$ after 350 cycles at 0.1 A $g^{-1}$ | |
| [36] | S@$Mn_1$-PNC | 1M $NaClO_4$ in EC/PC+5%FEC | 87 | 49 | 567 $mAhg^{-1}$/ 5 A | 344 mAh $g^{-1}$ after 3000 cycles at 2 A $g^{-1}$ | This work |
| [37] | S@ $Fe_1$-PNC | 1M $NaClO_4$ in EC/PC+5%FEC | 81 | 44 | 415 $mAhg^{-1}$/ 5 A | 720 mAh $g^{-1}$ after 120 cycles at 0.2 A $g^{-1}$ | |
| [38] | S@ $Co_1$-PNC | 1M $NaClO_4$ in EC/PC+5%FEC | 67 | 28 | 313 $mAhg^{-1}$/ 5 A | 572 mAh $g^{-1}$ after 120 cycles at 0.2 A $g^{-1}$ | |
| [39] | S@ $Ni_1$-PNC | 1M $NaClO_4$ in EC/PC+5%FEC | 53 | 38 | 464 $mAhg^{-1}$/ 5 A | 428 mAh $g^{-1}$ after 120 cycles at 0.2 A $g^{-1}$ | |
| [40] | S@ $Cu_1$-PNC | 1M $NaClO_4$ in EC/PC+5%FEC | 55 | 29 | 346 $mAhg^{-1}$/ 5 A | 454 mAh $g^{-1}$ after 120 cycles at 0.2 A $g^{-1}$ | |
| [41] | S@ $Sn_1$-PNC | 1M $NaClO_4$ in EC/PC+5%FEC | 64 | 33 | 451 $mAhg^{-1}$/ 5 A | 544 mAh $g^{-1}$ after 120 cycles at 0.2 A $g^{-1}$ | |

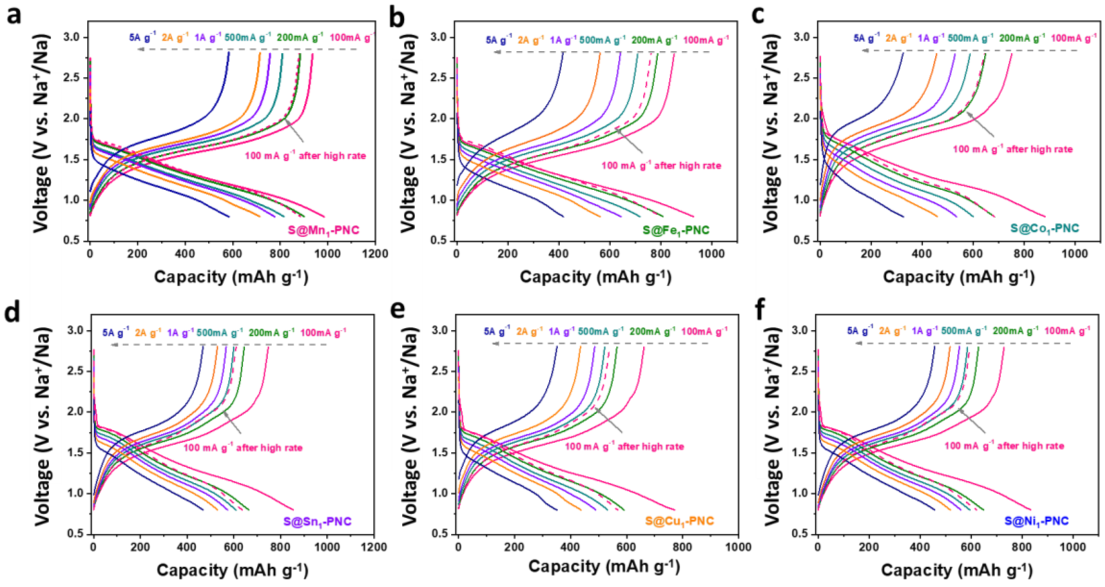


**Figure S18**. Charge-discharge curves at different current densities for (a) S@Mn1-PNC, (b) S@Fe1-PNC, (c) S@Co1-PNC, (d) S@Sn1-PNC, (e) S@Cu1-PNC, (f) S@Ni1-PNC.

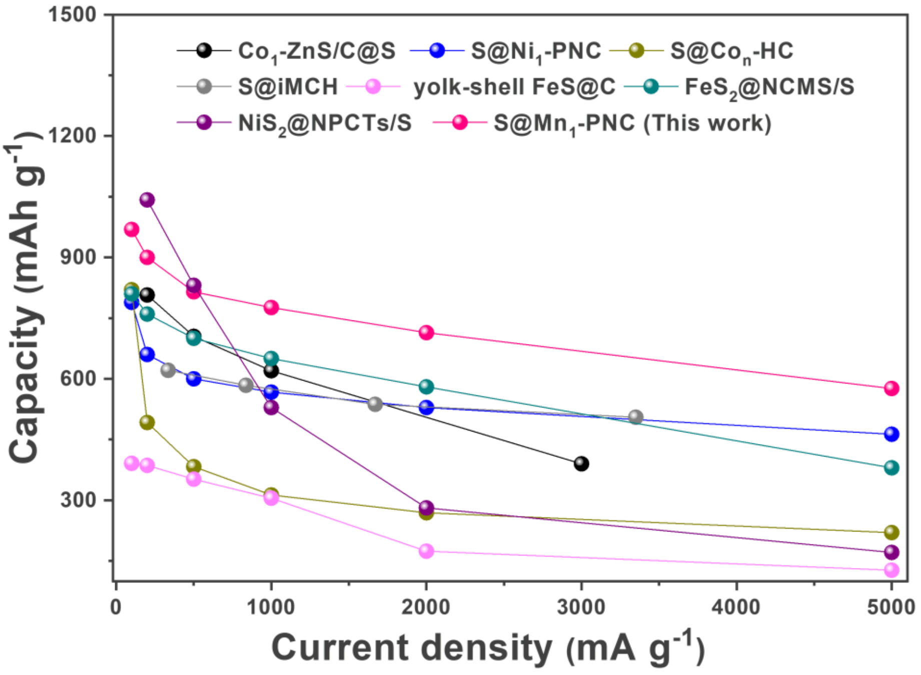


**Figure S19**. Rate performances of S@Mn1-PNC and S@Ni1-PNC cells compared with previous reports in the literature.

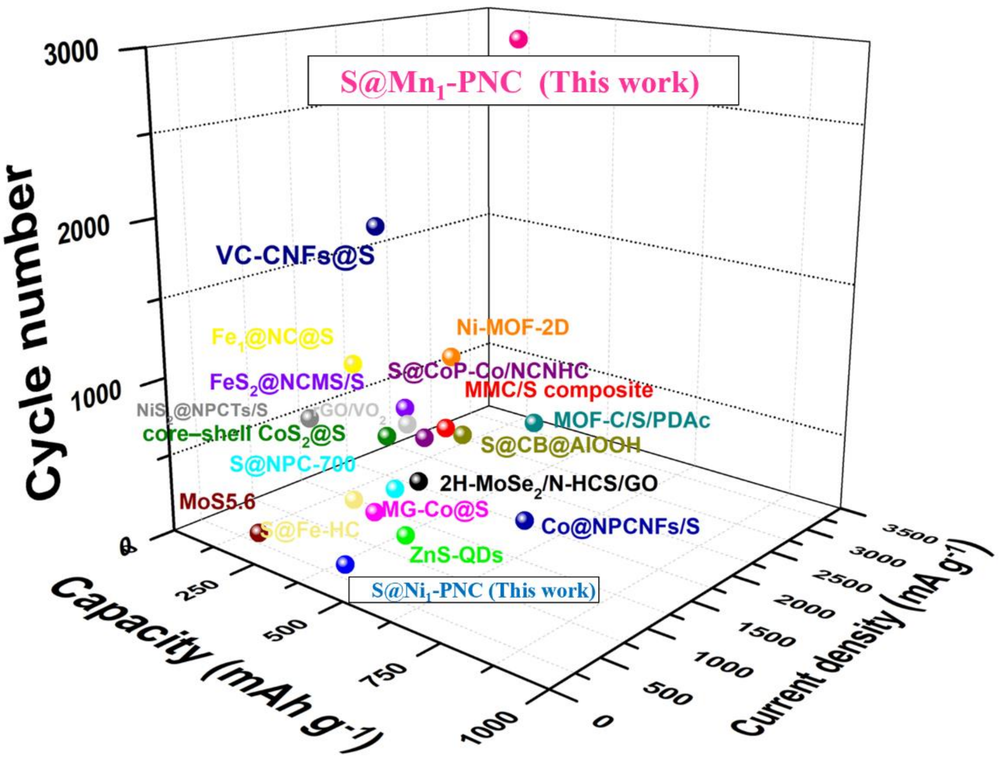


**Figure S20**. Cycling performance of S@Mn1-PNC compared with previous counterparts.

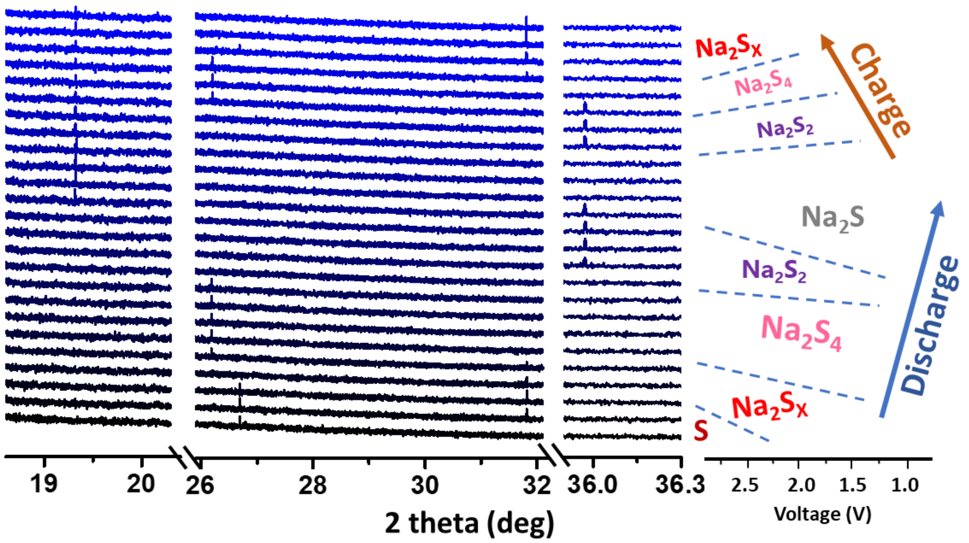


**Figure S21**. *In-situ* synchrotron-based XRD patterns of S@Ni1-PNC.

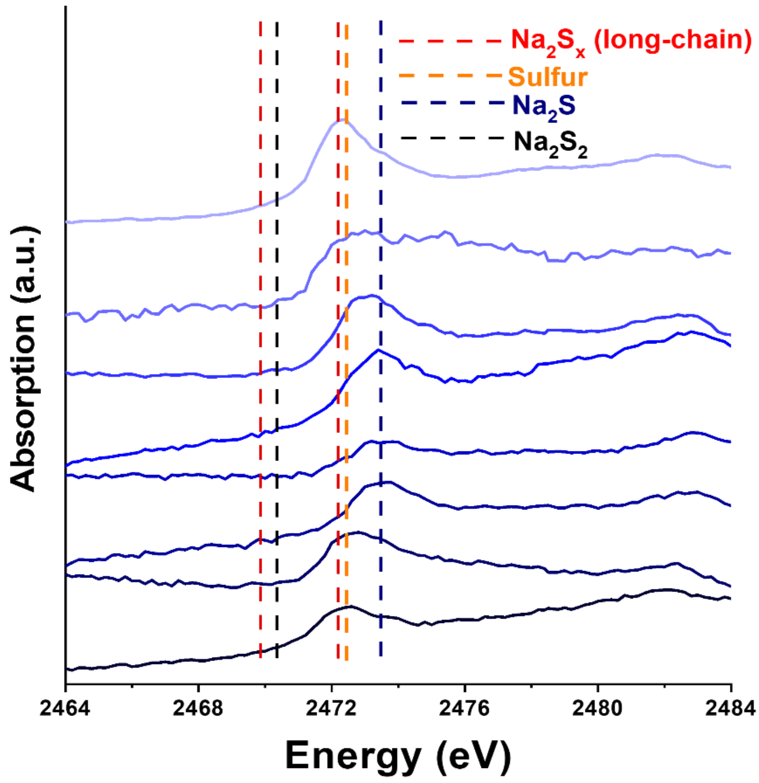


**Figure S22**. *Ex-situ* X-ray absorption spectra of S for S@Ni1-PNC during the initial cycle.

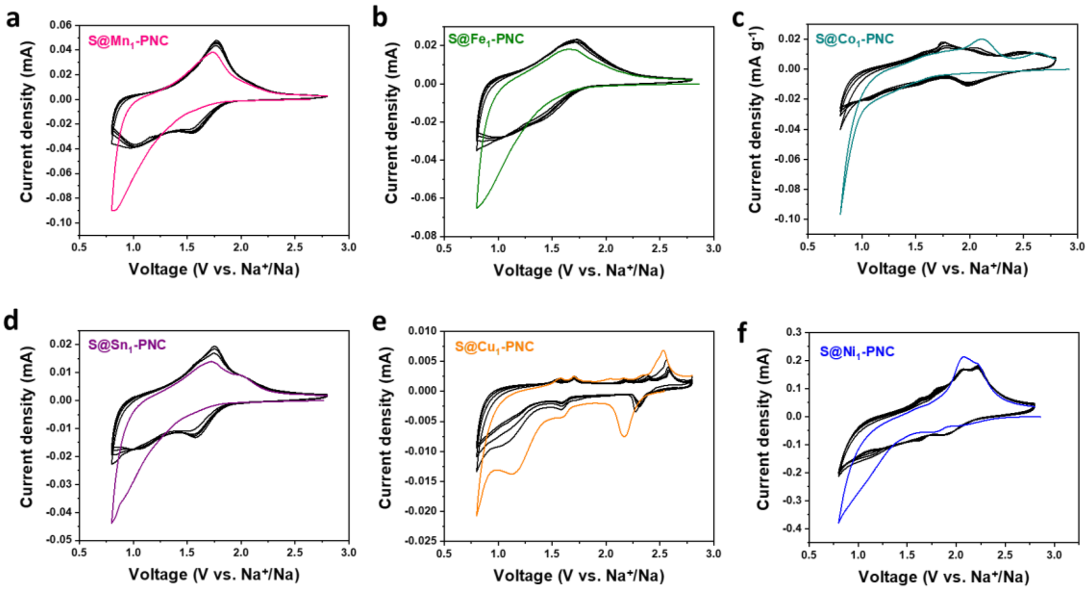


**Figure S23**. The first, second, and fifth cycle CV curves for (a) S@Mn1-PNC, (b) S@Fe1-PNC, (c) S@Co1-PNC, (d) S@Sn1-PNC, (e) S@Cu1-PNC, (f) S@Ni1-PNC.

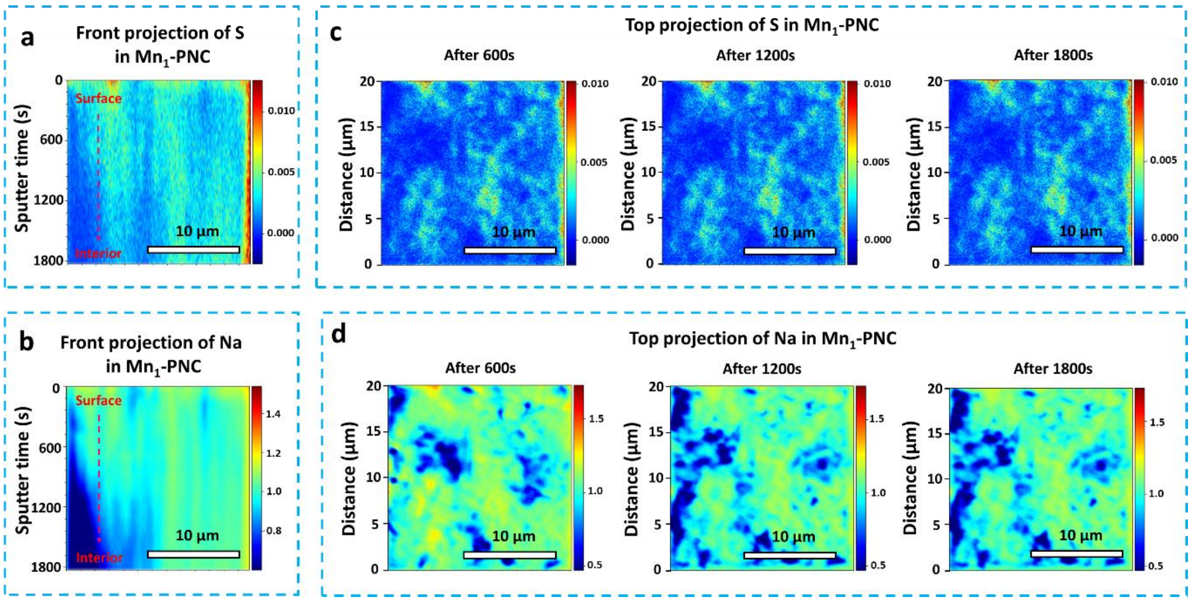


**Figure S24**. Time of flight-secondary ion mass spectroscopy (TOF-SIMS) images of the S@Mn1-PNC electrode after 50 cycles, showing the front projections of (a) S, (b) Na, and the top projections of (c) S and (d) Na.

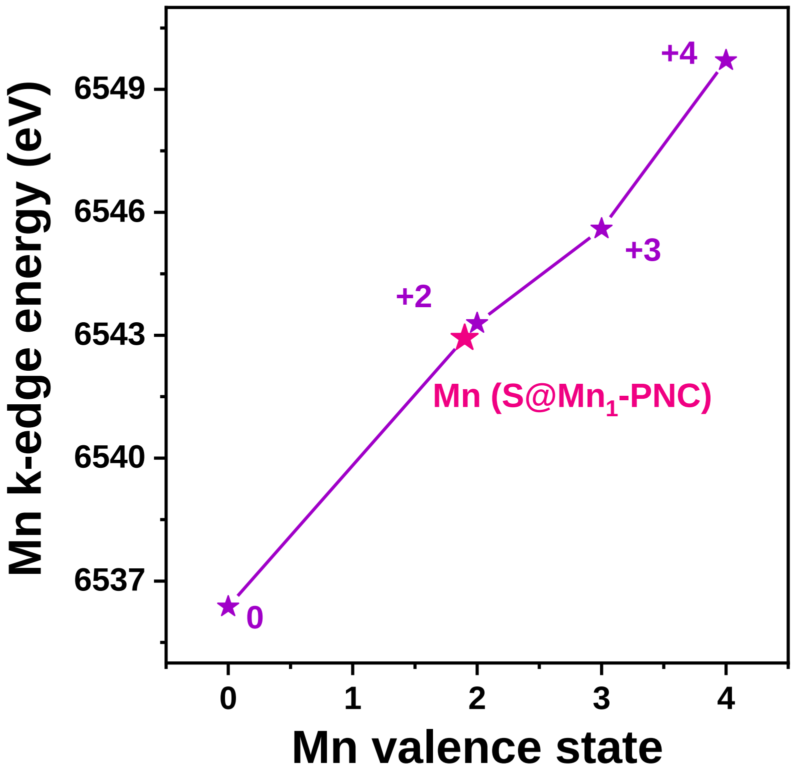


**Figure S25**. Valences of single Mn atoms in S@$Mn_1$-PNC according to XANES of the Mn K-edge.

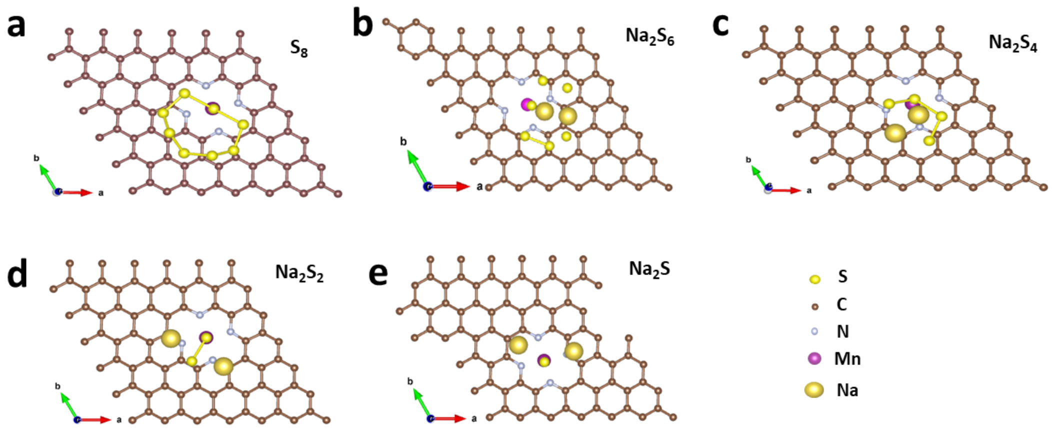


**Figure S26**. The absorption configurations of $Mn_1$ to (a) $S_8$, (b) $Na_2S_6$, (c) $Na_2S_4$, (d) $Na_2S_2$, (e) $Na_2S$.

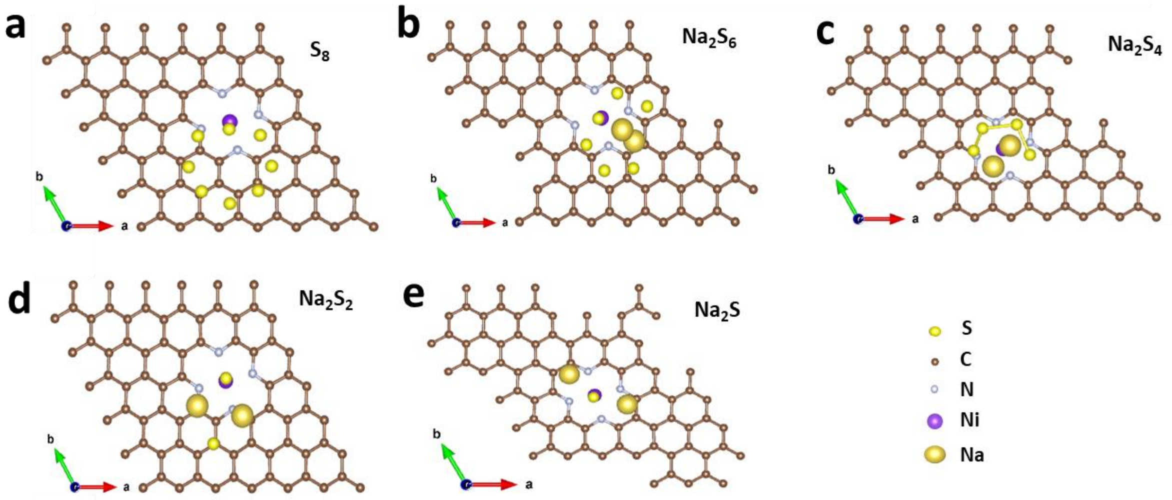


**Figure S27**. (a) The absorption configurations of $Ni_1$ to (a) $S_8$, (b) $Na_2S_6$, (c) $Na_2S_4$, (d) $Na_2S_2$, (e) $Na_2S$.

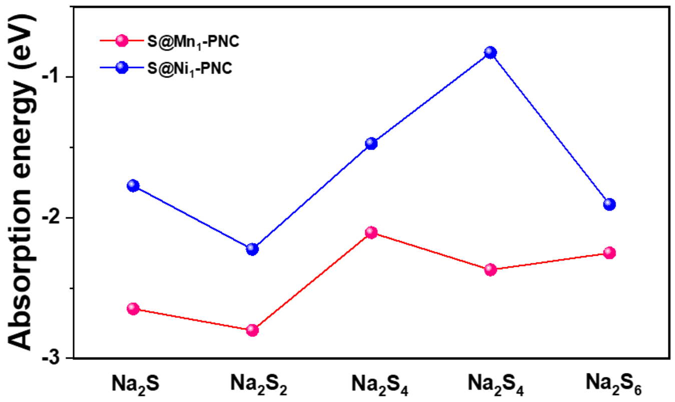


**Figure S28**. The energy absorption maps for the active sites of $Mn_1$ and $Ni_1$ with respect to polysulfides.

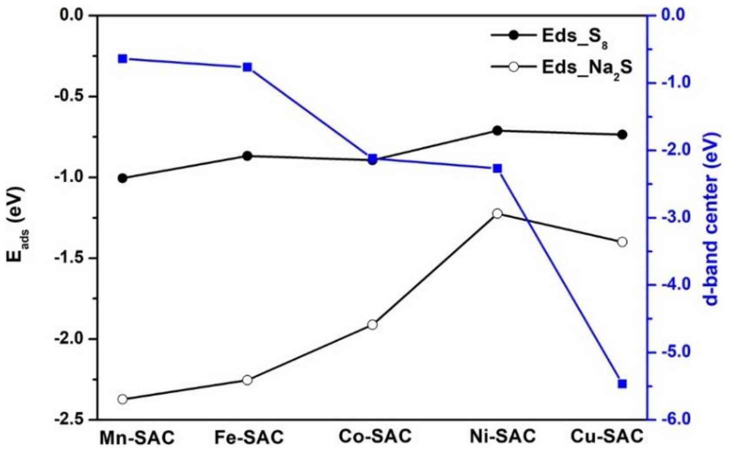


**Figure S29**. Correlation plots of adsorption energy and the *d*-band centre in five optimized single atom sites.

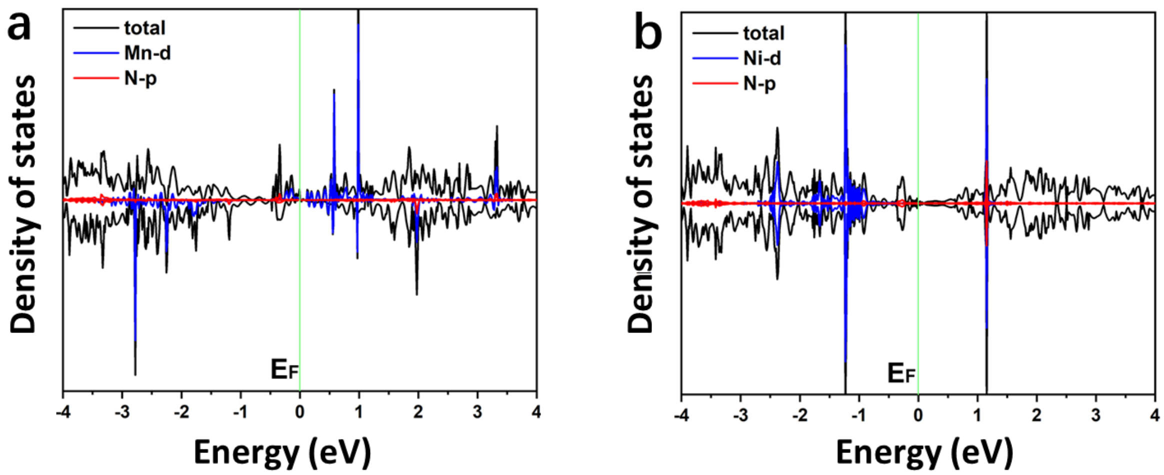


**Figure S30**. The density of states (DOS) of both (a) S@$Mn_1$-PNC and (b) S@$Ni_1$-PNC surfaces.

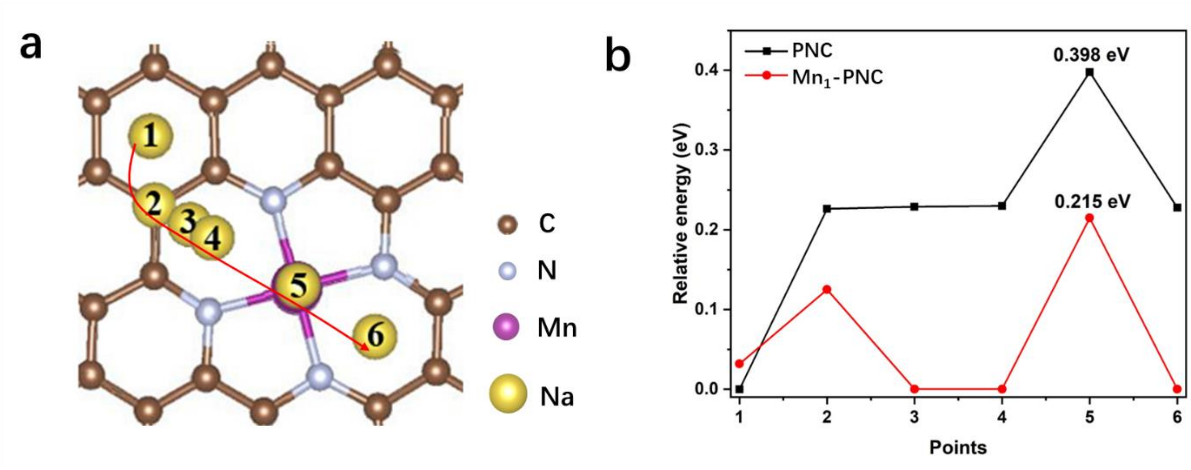


**Figure S31**. **Relative energy of Na ion diffusion on $Mn_1$-PNC and PNC**. (a) Schematic model of $Na^+$ diffusion path on $Mn_1$ anchored matrix. (b) the relative energy of sodium ion diffusion when $Mn_1$ is anchored on the matrix.

References


1 Yan, Z. *et al.* A High-Kinetics Sulfur Cathode with a Highly Efficient Mechanism for Superior Room-Temperature Na-S Batteries. *Adv. Mater.* **32**, 1906700 (2020).

2 Zhang, B.-W. *et al.* Atomic cobalt as an efficient electrocatalyst in sulfur cathodes for superior room-temperature sodium-sulfur batteries. *Nat. Commun.* **9**, 4082 (2018).

3 Zhang, B.-W. *et al.* Long-Life Room-Temperature Sodium-Sulfur Batteries by Virtue of Transition-Metal-Nanocluster-Sulfur Interactions. *Angew. Chem. Int. Ed.* **58**, 1484-1488 (2019).

4 Liu, H. *et al.* Electrocatalyzing S Cathodes via Multisulfiphilic Sites for Superior Room-Temperature Sodium-Sulfur Batteries. *ACS Nano* **14**, 7259-7268 (2020).

5 Yan, Z. *et al.* Electrochemical release of catalysts in nanoreactors for solid sulfur redox reactions in room-temperature sodium-sulfur batteries. *Cell Rep. Phys. Sci.* **2**, 100539 (2021).

6 Lai, W.-H. *et al.* General Synthesis of Single-Atom Catalysts for Hydrogen Evolution Reactions and Room-Temperature Na-S Batteries. *Angew. Chem. Int. Ed.* **59**, 22171-22178 (2020).

7 Liu, H. *et al.* Understanding Sulfur Redox Mechanisms in Different Electrolytes for Room-Temperature Na-S Batteries. *Nanomicro Lett.* **13**, 121 (2021).

8 Liu, H. *et al.* Sustainable S cathodes with synergic electrocatalysis for room-temperature Na-S batteries. *J. Mater. Chem. A.* **9**, 566-574 (2021).

9 Liu, D. *et al.* Stable Room-Temperature Sodium-Sulfur Batteries in Ether-Based Electrolytes Enabled by the Fluoroethylene Carbonate Additive. *ACS Appl. Mater. Interfaces.* **14**, 6658-6666 (2022).

10 Mou, J. *et al.* Hierarchical porous carbon sheets for high-performance room temperature sodium-sulfur batteries: integration of nitrogen-self-doping and space confinement. *J. Mater. Chem. A.* **8**, 24590-24597 (2020).

11 Wang, Y.-X. *et al.* Achieving High-Performance Room-Temperature Sodium-Sulfur Batteries With S@Interconnected Mesoporous Carbon Hollow Nanospheres. *J. Am. Chem. Soc.* **138**, 16576-16579 (2016).

12 Carter, R. *et al.* A Sugar-Derived Room-Temperature Sodium Sulfur Battery with Long Term Cycling Stability. *Nano Lett.* **17**, 1863-1869 (2017).

13 Zhang, L. *et al.* Self-Assembling Hollow Carbon Nanobeads into Double-Shell Microspheres as a Hierarchical Sulfur Host for Sustainable Room-Temperature Sodium-Sulfur Batteries. *ACS Appl. Mater. Interfaces.* **10**, 20422-20428 (2018).
