# Supplementary material for: Understanding the charge transfer effects of single atoms for boosting the performance of Na-S batteries
Source: Nat Commun. 2024 Apr 18;15:3325. doi: 10.1038/s41467-024-47628-3 (PMC11026416; doi:10.1038/s41467-024-47628-3)
Supplement: Supplementary file 3 — Description of Additional Supplementary Files [file 41467_2024_47628_MOESM3_ESM.pdf]

Description of Additional Supplementary Files

Supplementary Movie 1.

The sodiation process of $S@Mn_1$-PNC in the initial cycle.

Supplementary Movie 2.

The desodiation process of $S@Mn_1$-PNC in the initial cycle.
